# Immunospecific Responses to Bacterial Elongation Factor Tu during *Burkholderia* Infection and Immunization

**DOI:** 10.1371/journal.pone.0014361

**Published:** 2010-12-17

**Authors:** Wildaliz Nieves, Julie Heang, Saja Asakrah, Kerstin Höner zu Bentrup, Chad J. Roy, Lisa A. Morici

**Affiliations:** 1 Department of Microbiology and Immunology, Tulane University School of Medicine, New Orleans, Louisiana, United States of America; 2 Division of Microbiology, Tulane National Primate Research Center, Covington, Louisiana, United States of America; Cairo University, Egypt

## Abstract

*Burkholderia pseudomallei* is the etiological agent of melioidosis, a disease endemic in parts of Southeast Asia and Northern Australia. Currently there is no licensed vaccine against infection with this biological threat agent. In this study, we employed an immunoproteomic approach and identified bacterial Elongation factor-Tu (EF-Tu) as a potential vaccine antigen. EF-Tu is membrane-associated, secreted in outer membrane vesicles (OMVs), and immunogenic during *Burkholderia* infection in the murine model of melioidosis. Active immunization with EF-Tu induced antigen-specific antibody and cell-mediated immune responses in mice. Mucosal immunization with EF-Tu also reduced lung bacterial loads in mice challenged with aerosolized *B. thailandensis*. Our data support the utility of EF-Tu as a novel vaccine immunogen against bacterial infection.

## Introduction

The bacterium *Burkholderia pseudomallei* is a Gram-negative, facultative intracellular bacillus and the causative agent of melioidosis, a serious emerging disease responsible for significant morbidity and mortality in Southeast Asia and Northern Australia [1]. Natural infection can occur through subcutaneous inoculation, ingestion, or inhalation of the organism. Clinical manifestations are nonspecific and widely variable, and may include acute septicemia, pneumonia, and chronic infection [2]. Mortality rates associated with severe *B. pseudomallei* infection approach 50% and can reach 80–95% in patients with septic shock despite antibiotic treatment [3], [4]. This is partially due to the innate antimicrobial resistance of *B. pseudomallei* as well as the intracellular niche of the organism [1], [5]. Thus, preventive measures such as active immunization are needed to reduce the morbidity and mortality associated with *B. pseudomallei* infection.

Previous immunization strategies that utilized heat-killed or live-attenuated *B. pseudomallei*, lipopolysaccharide (LPS), capsular polysaccharide (CPS), or protein-based (i.e. Type III secretion system (TTSS-3) or outer membrane proteins) subunits conferred variable degrees of protection against systemic challenge but have proved ineffective or have not been tested against aerosol infection [6]–[13]. In addition, vaccine preparations administered parenterally with aluminum hydroxide adjuvant elicit robust antibody and Type 2 immune responses against *B. pseudomallei* but are insufficient for complete protection [14]. Antibody responses alone are often deficient in providing sterile immunity against intracellular bacterial pathogens [15]. An ideal vaccine against *B*. *pseudomallei* will likely require the induction of a Type 1 cellular-mediated immune (CMI) response for complete efficacy as suggested from past immunization studies [9], [16]. Furthermore, the nasal associated lymphoid tissue (NALT) may represent a primary site of invasion by *B. pseudomallei* [17]. Vaccine strategies that target the mucosal surface and induce Type 1 responses may therefore provide enhanced protection against aerosol infection with *B. pseudomallei*.

Use restrictions associated with *B*. *pseudomallei*, a biosafety level three select agent, have hampered vaccine development. We therefore employed an immunoproteomic approach to identify a number of novel immunoreactive proteins in *B. thailandensis* that have potential for use as subunit vaccines against inhalational *B. pseudomallei* infection. *B. thailandensis* shares 94% identity with *B. pseudomallei* at the amino acid level and has served as a useful surrogate for *B. pseudomallei* in multiple studies [18]–[22]. Here we report a novel role for bacterial Elongation Factor-Tu (EF-Tu) as a vaccine immunogen and demonstrate its ability to elicit antibody and CMI responses in immunized mice. We also test the protective capacity of EF-Tu immunization in a *B. thailandensis* aerosol challenge model [20], [21].

## Materials and Methods

### Ethics Statement

All experimental procedures involving animals were approved (protocol numbers 4042E and 4048D) and performed under strict compliance with the guidelines established by Tulane University Health Sciences Center and Tulane National Primate Research Center Institutional Animal Care and Use Committees.

### Two-dimensional gel electrophoresis

Two-dimensional (2D)- gel electrophoresis was performed using 100 µg of *B. thailandensis* whole cell lysate solubilized in 7 M urea, 2 M thiourea, 4% (w/v) 3-[3-(cholamidopropyl)dimethylammonio]-1-proanesulphonate (CHAPS), 20% glycerol, 30 mM Tris, pH 8.5. Fifty µg of the crude lysate was used to rehydrate an 18 cm IPG strip, pH 3–10 non-linear (NL) overnight. The following day, the proteins in the rehydrated strip were subjected to isoelectric focusing at 50 µA/strip. The strip was then equilibrated 15 min with 20 mg/ml dithiothreitol (DTT) and 25 mg/ml iodoacetamide before loading onto a 12.5% SDS-polyacrylamide gel (Invitrogen). The gel was run for 30 min at 5 Watts/gel and then for 5 hr at 18 Watts/gel. Western blot was performed as described below with a few modifications: the membrane was blocked with 5% skim milk in TBS containing 0.05% Tween 20 (TBST); a 1∶200 dilution of polyclonal serum from New Zealand White rabbits that were immunized subcutaneously with irradiated *B. mallei* ATCC 23344 (kindly provided by Dr. David DeShazer, USAMRIID) was used as the primary antibody; and a 1∶2000 dilution of a goat anti-rabbit HRP-conjugated antibody (BD Pharmingen, San Diego, CA) was used as the secondary.

### Matrix assisted laser desorption ionization time of flight (MALDI-TOF) mass spectrometry

MALDI-TOF analysis was performed on a 4700 Proteomics Analyzer MALDI-TOF-TOF (Applied Biosystems, Foster City, CA). An averaged simple mass spectrum and tandem mass spectra from the five most abundant peptides (excluding trypsin autolysis) of each sample were acquired and manually inspected in Data Explorer. Global Proteome Server (Applied Biosystems) was utilized to search the bacteria of Uniprot protein database. One missed cleavage per peptide was allowed, and the fragment ion mass tolerance window was set to 100 ppm. A protein hit with a total score of 75 or higher, with at least one peptide over 20, was considered a likely match. Protein similarities were obtained using Basic Local Alignment Search Tools (BLAST) (http://www.ncbi.nlm.nih.gov/BLAST) and the NCBI non-redundant database.

### Cloning, expression, and purification of EF-Tu

Based on the published genome sequence of *B. pseudomallei* strain K96243, the complete open reading frame (ORF) of EF-Tu was PCR amplified from *B. pseudomallei* strain 286 genomic DNA (BEI Resources, Manassas, VA) using the forward primer 5′-GCATGCGCCAAGGAAAAGTTTGAGCGGACC-3′ and the reverse primer 5′- AAGCTTTTACTCGATGATCTTGGCGACGACG -3′ which produces SphI and HindIII (underlined) sites at the 5′- and 3′- ends of the EF-Tu ORF respectively. The fragment was ligated into the multi-cloning site of the protein expression vector pQE30 (Qiagen, Valencia, CA) containing an N-terminal 6X-histidine tag, and transformed into *E.coli* strain DH-5α for automated sequencing using the pQE forward and reverse sequencing primers (Qiagen). The cloned EF-Tu from strain 286 shares 100% amino acid sequence identity with EF-Tu from *B. pseudomallei* strain K96243 (Uniprot/Swiss prot #Q63PZ6) and *B. thailandensis* E264 (Uniprot/Swiss prot #Q2SU25) and 79.4% identity with *E. coli* K12 (Uniprot/Swiss prot #P0CE48). For over-expression of the EF-Tu protein, the construct was transformed into *E. coli* strain M15 (Qiagen) and transformants were cultured overnight at 37°C in Luria-Bertani (LB) broth supplemented with ampicillin (100 µg/ml) and kanamycin (50 µg/ml). A 1∶100 dilution was used to inoculate fresh LB broth supplemented with ampicillin (50 µg/ml) and kanamycin (25 µg/ml) and allowed to grow to mid-log phase before induction with 1 mM isopropyl-β-D- thiogalactoside (IPTG) for 4 hr. Cells were harvested by centrifugation and the cell pellet was stored at −80°C overnight. Cells were resuspended in lysis buffer (50 mM NaH_2_PO_4_, 300 mM NaCl, 10 mM imidazole), and sonicated three times for 30 sec. Supernatant containing recombinant EF-Tu (rEF-Tu) protein was collected after centrifugation, and simple batch purification was achieved using Ni-NTA agarose beads (Qiagen) under native conditions. Agarose beads were washed three times with buffer containing 20 mM imidazole, five times with 0.5% amidosulfobetaine-14 (ASB-14) to remove lipopolysaccharide (LPS), five times with 20 mM Tris-HCl, and eluted with 250 mM imidazole. Eluted protein fractions were concentrated by centrifugation (Amicon, MW cutoff 10,000 kDa), and imidazole was removed by buffer exchange with LPS-free water. LPS contamination was determined to be less than 25 EU/ml using the limulus amebocyte lysate (LAL) assay (Lonza, Switzerland). Protein concentration was determined using the Bradford protein assay (BioRad).

### Total membrane protein extraction, SDS-PAGE, and Western blot

A single colony of either *B. thailandensis or E. coli* was used to inoculate LB broth and incubated overnight. Each culture was freshly diluted 1∶100 into LB broth the next morning. The bacterial cells were grown to log-phase and harvested by centrifugation (6,000×*g*, 10 min, 4°C). The bacterial pellet was resuspended in 1/50^th^ volume of 4-(2-hydroxyethyl)-1-piperazineethanesulfonic acid (HEPES) buffer (10 mM, pH 7.4). Lysozyme was added at a final concentration of 10 mg/ml and incubated for 20 min at room temperature. The bacterial suspension was sonicated five times (50-Watts) for 30 sec each on ice. Benzonase (Novagen, Gibbstown, NJ) was added at a final concentration of 1 µg/ml, and the lysate was incubated for 30 min at room temperature. Intact cellular debris was removed by centrifugation (12,000×*g*, 10 min, 4°C). A sample of the supernatant consisting of the whole cell lysate was stored at −80°C until use. The remaining supernatant was centrifuged (50,000×*g*, 60 min, 4°C), and the resulting pellet was resuspended in 0.5% Sarkosyl (Sigma) and incubated 30 min at room temperature. The suspension consisting of total membrane proteins (both inner and outer membrane) was aliquoted and stored at −80°C until use.

Sodium dodecyl sulphate polyacrylamide gel electrophoresis (SDS-PAGE) was performed using a 4–20% polyacrylamide gel (Bio-Rad). rEF-Tu or proteins from whole cell lysate or total membrane fractions of either *B*. *thailandensis or E. coli* were separated under reducing conditions, and the proteins were subsequently transferred to nitrocellulose membranes. The membranes were blocked with 1.5% BSA in TBST overnight at 4°C and then washed twice with TBST. The membranes were then incubated overnight at 4°C with pooled sera (1∶200 dilution) from rEF-Tu immunized mice; pooled sera (1∶200 dilution) obtained from mice 2 weeks after intraperitoneal (i.p.) challenge with 10^7^ cfu *B. thailandensis* strain E264 (American Type Culture Collection (ATCC), Manassas, VA); pooled (pre-immune) sera (1∶200 dilution) from naïve mice; or with a monoclonal antibody (1∶1000 dilution) to the β subunit of *E. coli* RNA polymerase (Neoclone, Madison, WI). The RNA polymerase antibody did not recognize *B. thailandensis* and was therefore used only on *E. coli* cellular fractions to determine the purity of total membrane preparations. The membranes were subsequently washed three times with TBST and incubated with goat anti-mouse HRP-conjugated secondary antibody (1∶1000 dilution) (Thermo Scientific Pierce, Rockford, IL) for 1 hr at room temperature. The membranes were washed twice with TBST and developed with Opti-4CN Substrate (BioRad, Hercules, CA).

### Outer membrane vesicle (OMV) preparation

OMVs were prepared as previously described [23], [24] with minor modifications. *B. pseudomallei* strain 1026b (BEI Resources) was grown in LB broth at 37°C until late log phase (16–18 hr). The intact bacteria were pelleted by centrifugation at 6,000×g for 10 min at 4°C, and the supernatant was removed and filtered through a 0.22 µm polyethersulfone (PES) filter (Millipore) in order to remove any remaining bacteria or large bacterial fragments. To ensure the supernatant was free of viable bacteria, 1 mL of supernatant was streaked onto PIA agar and incubated 48–72 hrs at 37°C. The remaining filtered supernatant was incubated at 4°C. OMVs were harvested by slowly adding 1.5 M solid ammonium sulfate (Fisher Scientific) while stirring gently and incubated overnight at 4°C. The OMVs were harvested by centrifugation at 11,000×g for 20 min at 4°C. The resulting pellet, consisting of crude vesicles, was resuspended in 45% OptiPrep (Sigma) in 10 mM HEPES/0.85% NaCl, pH 7.4, filter sterilized through a 0.22 µm PES filter and layered at the bottom of a centrifuge tube. An OptiPrep gradient was prepared by slowly layering 40%, 35%, 30%, 25%, and 20% OptiPrep in HEPES-NaCl (w/v) over the crude OMV preparation. Membrane vesicles were collected by ultracentrifugation at 111,000×g for 2 hr at 4°C. Equal fractions were removed sequentially from the top and stored at 4°C. To determine the purity of the fractions, 250 µl of each was precipitated with 20% (w/v) Tri-chloroacetic acid (TCA). The resulting pellet was resuspended in 10 µl phosphate buffered saline (PBS) and 10 µl Laemmli loading buffer (Bio-Rad), boiled for 10 min and loaded onto an SDS-PAGE polyacrylamide gel (4–20% Mini Protean, Bio-Rad) run at 200 V. The working OMV preparation was recovered by pooling the peak fractions (those containing the least amount of insoluble fragments and contaminants) in 50 mM HEPES, pH 6.8 followed by centrifugation at 111,000×g for 2 hr at 4°C. The resulting pellet containing OMVs was resuspended in LPS-free water (Lonza) and stored at −20°C. OMVs were quantified with a Bradford Protein Assay (Bio-Rad). Cryo-Transmission Electron Microscopy was performed using a JEOL 2010 transmission electron microscope to visually confirm the presence of OMVs.

### Animals

Female BALB/c mice 8- to 10-weeks-old were purchased from Charles River Laboratories (Wilmington, MA) and maintained 5 per cage in polystyrene microisolator units under pathogen-free conditions. Animals were fed sterile rodent chow and water *ad libitum* and allowed to acclimate 1 week prior to this study. Mice were euthanized by carbon dioxide overdose.

### Bacterial challenges

#### Intraperitoneal (i.p.)

Prior to murine challenge, *B. thailandensis* was freshly grown from frozen glycerol stock in LB broth overnight and freshly diluted 1∶100 into LB broth the next morning. The bacteria were grown to log-phase and harvested by centrifugation and diluted into 0.9% NaCl to 1×10^8^ colony forming units (cfu)/ml. Each mouse (N = 6) was administered 100 µl of bacteria (10^7^ cfu) via the i.p. route. Mice were monitored for symptoms of illness twice daily for 14 days and survivors were euthanized at the end of study. Blood samples were collected via cardiac puncture following euthanasia. Blood was allowed to clot for 30 min at room temperature and then centrifuged at 2300×*g*; serum was collected and stored at −80°C until use.

#### Aerosol

BALB/c mice were challenged with 5×10^5^ cfu (∼LD_50_) of *B. thailandensis* using a nose-only inhalation exposure chamber as previously described [20], [21]. Mice were euthanized at 24 hr post-challenge, and lungs were collected for determination of lung bacterial cfu.

### Immunizations

BALB/c mice (N = 70) were primed subcutaneously (s.c.) on day 0 with 25 µg of purified rEF-Tu in LPS-free water adsorbed 1∶1 with aluminum hydroxide adjuvant (Alhydrogel 2%, Brenntag, Germany) in a final volume of 100 µl or intranasally (i.n.) with 25 µg rEF-Tu in LPS-free water admixed with 5 µg CpG oligodeoxynucleotide (ODN) 1826 adjuvant (Coley, Wellesley, MA) in a final volume of 9 µl/ nostril. Prior to intranasal immunization, mice were anesthetized via the i.p. route with 0.88 mg/kg ketamine/xylazine in saline in a final volume of 100 µl. Mice were boosted on day 21 with the same formulations using a homologous (s.c. + s.c. or i.n. + i.n.) or heterologous (s.c. + i.n.) prime/boost strategy.

### Analysis of antibody response

Blood samples from immunized and naive mice were collected via cardiac puncture following euthanasia for determination of rEF-Tu specific serum antibody concentration. Blood was allowed to clot for 30 min at room temperature and then centrifuged at 2300×*g*; serum was collected and stored at −80°C until assayed. Bronchoalveolar lavage (BAL) fluid was collected for determination of rEF-Tu specific BAL antibody concentration. BAL fluid was obtained by exposing the trachea and making a small incision into which an 18-gauge needle was inserted and secured. The lungs were repeatedly lavaged by slowly injecting and withdrawing 1 ml of phosphate buffered saline (PBS) supplemented with Complete protease inhibitor cocktail (Roche Laboratories, Mannheim, Germany). BAL fluid was stored at −80°C until assayed. The concentrations of serum and BAL fluid rEF-Tu-specific total IgG, IgG1, IgG2a, and IgA were analyzed by enzyme-linked immunosorbent assay (ELISA). Ninety-six-well microtiter plates were coated with 0.5 µg per well of purified rEF-Tu in coating buffer (0.1 M sodium bicarbonate, 0.2 M sodium carbonate) and incubated overnight at 4°C. The plates were washed three times with PBS containing 0.05% Tween-20 (PBST). For measurement of IgA, plates were additionally blocked with 2% BSA for 1 hr followed by three washes with PBST. All plates were incubated with two-fold serial dilutions of sera or BAL samples for 2 hr at room temperature. Plates were washed three times with PBST and then incubated with either alkaline phosphatase (AP)-conjugated rat anti-mouse IgG, IgG1, IgG2a (1∶300 dilution in PBST) (BD Pharmingen) or AP-conjugated goat-anti-mouse IgA (1∶2000) (Invitrogen) for 1 hr at room temperature. At the end of the incubation, the plates were washed three times with PBST and developed with SIGMA*FAST* p-Nitrophenyl phosphate tablets (Sigma, St. Louis, MO) dissolved in diethanolamine buffer (1 mg/ml). After 15–30 min of incubation, reaction solutions were stopped with 2 M NaOH and read at 405 nm using a µQuant microplate reader and analyzed with Gen5 software (BioTek, Winooski, VT). Antibody concentrations were determined by non-linear regression from a standard curve of mouse myeloma IgG1, IgG2a, and IgA (Sigma) serially diluted as a standard on each ELISA plate [25]. The results obtained are expressed as the mean concentration ± standard error of the mean (SEM).

### Antigen restimulation assay

Restimulation assays were performed with splenocytes from immunized and naïve mice for analysis of T cell responses. Spleens were removed aseptically and single-cell splenocyte suspensions from each mouse were obtained by passing the spleens through sterile 40 µm cell strainers (Fisher Scientific; Pittsburgh, PA). Cells were washed twice with wash buffer (Advanced RPMI 1640 medium supplemented with 1% fetal bovine serum (FBS) and 1% antibiotic-antimycotic) (Invitrogen). Cell pellets were resuspended in wash buffer and layered onto Histopaque-1119 (Sigma) for splenic mononuclear leukocyte isolation by centrifugation at 300×*g* for 15 min. Leukocytes were recovered at the interface and washed twice with wash buffer and resuspended in Advanced RPMI 1640 medium supplemented with 10% FBS and 1% antibiotic-antimycotic. Cells were plated in a 96-well microtiter plate at 4×10^5^ cells/well. Cell cultures were stimulated with 1 µg of rEF-Tu, 1 µg concanavalin A (ConA) (Sigma), or left unstimulated as negative controls. The cultures were incubated at 37°C in 5% CO_2_, and cell culture supernatants from each treatment group were collected after 72 hr and stored at −80° until use.

### CFU recovery

Lung tissue homogenates were used to determine bacterial burden in aerosol-infected mice. Lungs were aseptically removed, weighed, and individually placed in 1 ml 0.9% NaCl and homogenized with a Power Gen 125 (Fisher Scientific). Ten-fold serial dilutions of lung homogenates were plated on LB agar. Colonies were counted after incubation for 2–3 days at 37°C and reported as cfu per gram of tissue.

### Statistical analyses

All analyses were performed using GraphPad Prism version 5.0 (GraphPad Software, Inc., La Jolla, CA). Statistical analysis of cytokine production was performed using a two-way ANOVA, and analyses of antibody concentrations and bacterial burdens were performed using the Mann-Whitney test. Values of *P*<0.05 were considered statistically significant.

## Results

### Identification of EF-Tu as a potential vaccine candidate for B. pseudomallei

**Figure 1:**
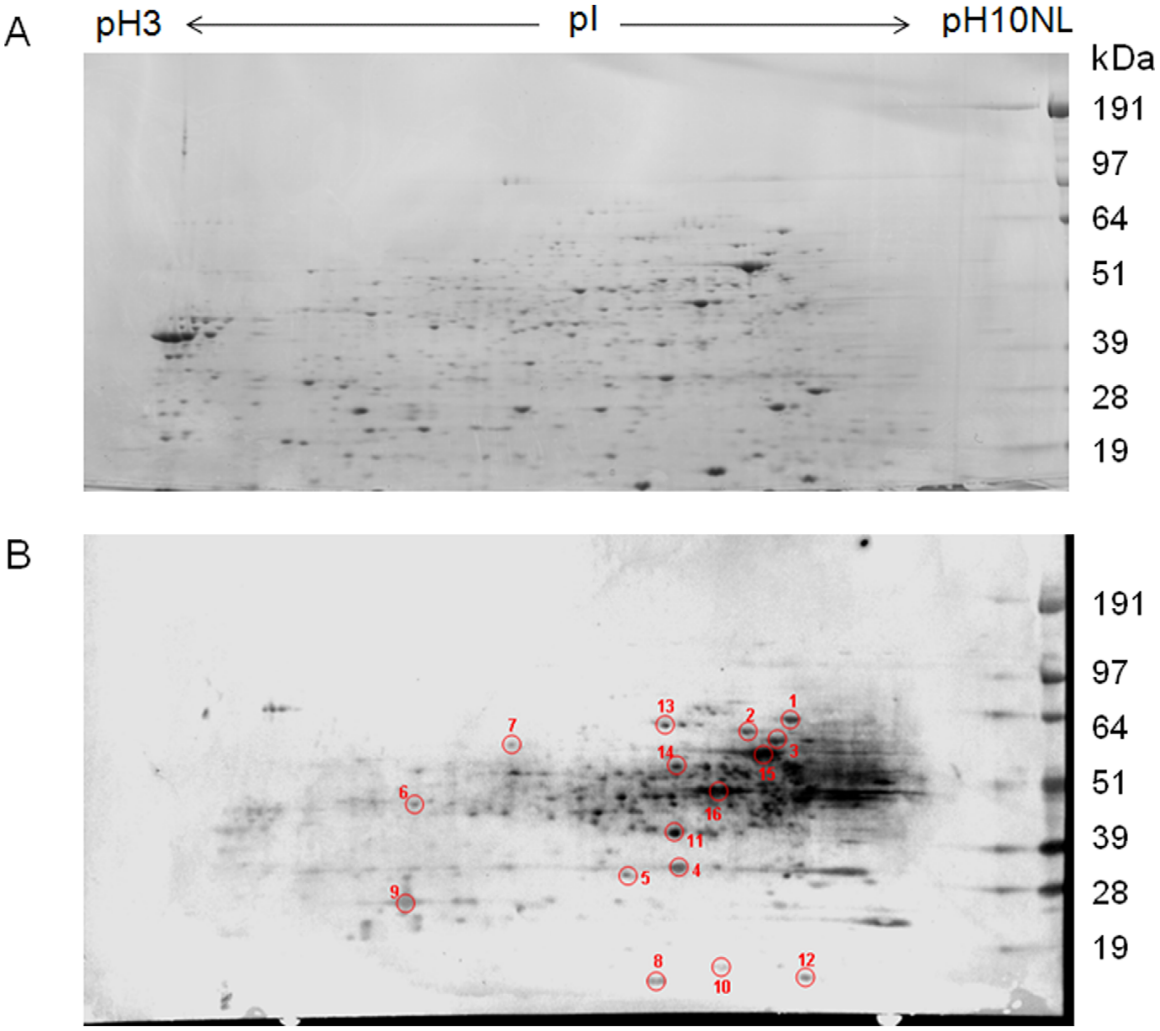
*B. thailandensis* whole cell lysate separated by two-dimensional gel electrophoresis. (A) SYPRO-ruby stained gel (B) Western blot performed using rabbit anti-*B. mallei* polyclonal sera (1∶200 dilution), followed by HRP-conjugated goat anti-rabbit IgG (1∶2000) and detected with Opti-4CN substrate (BioRad).

We employed an immunoproteomic approach [26] to identify novel immunogenic *Burkholderia* proteins that could be further screened for their ability to elicit both antibody and CMI responses. At that time, antisera against *B. pseudomallei* was not available to us. Therefore, pooled antisera from *B. mallei*-immunized rabbits was used to probe a *B. thailandensis* whole cell lysate that was separated by 2D-gel electrophoresis (Figure 1A). We hypothesized that proteins shared by *B. mallei*, *B. pseudomallei*, and *B*. *thailandensis* could be detected by this approach due to the extensive homology between the three species [19]. The immunoblot revealed more than 100 immunoreactive proteins of which we randomly selected 16 spots for identification by MALDI-TOF mass spectrometry (Figure 1B). None of the selected spots were detected using antisera from naïve rabbits (not shown). Eleven proteins were successfully identified and share 96–100% amino acid identity among the three *Burkholderia* species (Table 1). Three of the proteins, EF-Tu, AhpC, and DnaK, were previously recognized as potential *B*. *pseudomallei* antigens using a similar approach with human convalescent sera [27]. Surprisingly, one of the immunogenic proteins identified by both studies was EF-Tu. EF-Tu is best known for its role in bacterial protein synthesis, functioning as a GTPase to catalyze the transfer of aminoacyl-tRNAs to the ribosome [28]. However, compelling evidence supports additional functions for EF-Tu, including roles as a bacterial adhesin and invasin for several pathogenic bacteria [29]–[32].

**Table 1:** Immunoreactive *B. thailandensis* proteins identified by MALDI-TOF mass spectrometry.

Sample Number	Protein	Accession Number	Function	Theoretical MW (kDa)	Theoretical p*I*	% identity to *B. pseudomallei* K96243	% identity to *B. mallei* ATCC 23344
**1**	**DnaK**	83721009	heat shock protein	69.70	4.96	98%	98%
**2**	HtpG	83720569	heat shock protein	70.97	5.17	99%	99%
**3**	30S ribosomal protein	83719745	translation	62.26	5.08	100%	100%
**4**	3-oxoadipate CoA-succinyl transferase	83721125	metabolism	33.16	9.06	98%	98%
**5**	**AhpC**	83721537	peroxidase	23.81	5.61	98%	98%
**6**	*						
**7**	*						
**8**	hypothetical proteinBTH_110718	83717445		11.87	5.64	not present	not present
**9**	OmpW	83719376	outer membrane protein	22.71	8.6	96%	96%
**10**	cpn10	83719093	heat shock protein	10.48	5.33	99%	99%
**11**	CmaB	83717262	translation	35.36	5.44	97%	not present
**12**	ribosomal protein L7	83719193	translation	12.52	4.9	97%	98%
**13**	*						
**14**	*						
**15**	*						
**16**	**EF-Tu**	83721154	translation	42.86	5.36	100%	100%

Putative function and % identity to *B*. *pseudomallei* K96243 and *B. mallei* ATCC 23344 at the amino acid level are shown. Proteins in bold were also identified by Harding *et al*. [27] using a similar approach. * unidentified by mass spectrometry. “Not present” indicates that no known ortholog is annotated in the NCBI genomic database.

### Burkholderia EF-Tu is membrane-associated and recognized during natural infection

**Figure 2:**
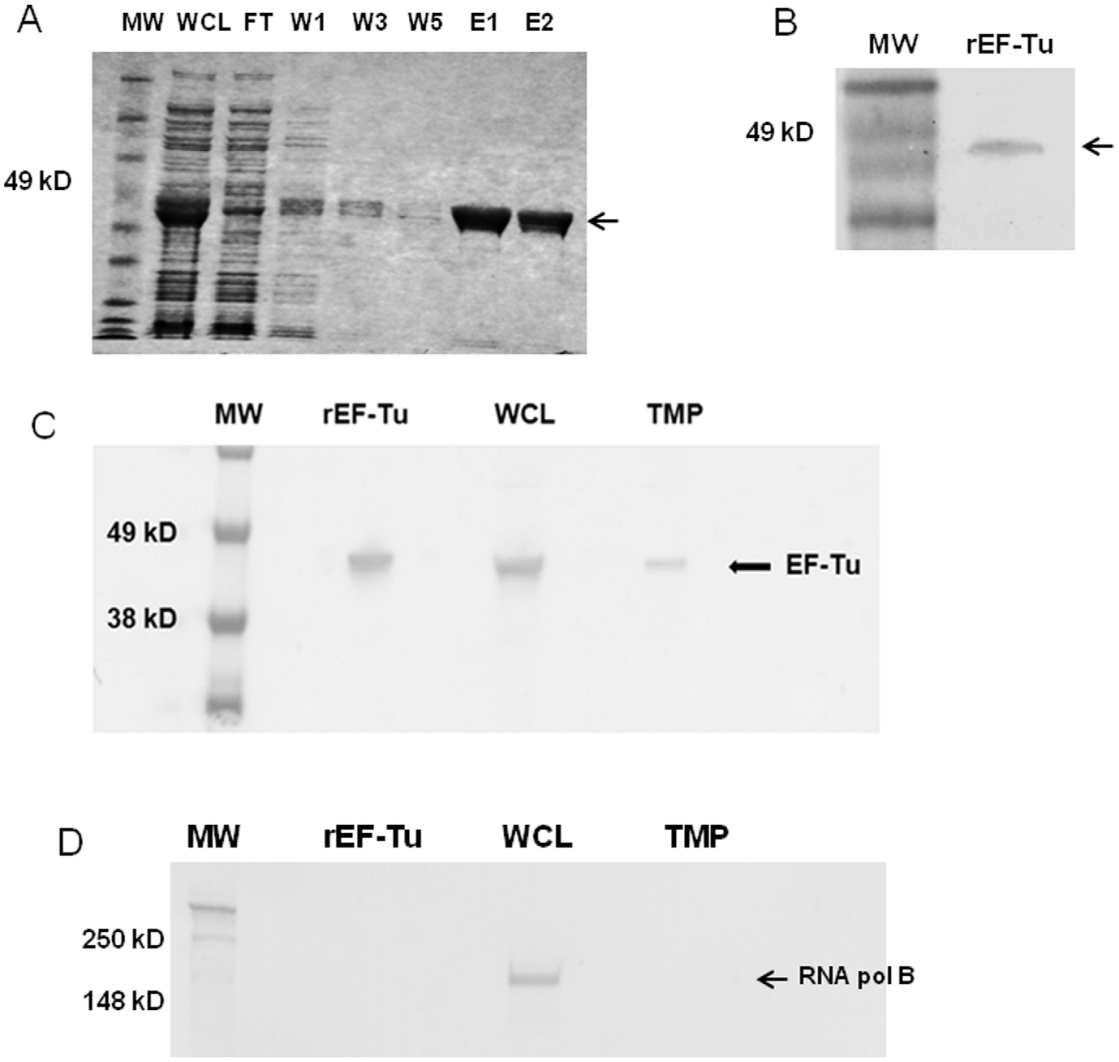
Immunogenicity of EF-Tu during infection and immunization. (A) Coomassie stained gel of rEF-Tu affinity purified under native conditions by Ni-NTA agarose batch purification. MW = BenchMark Pre-stained molecular weight ladder; Whole-cell lysate (WCL) = 5 µg; Flow-through (FT) = 5 µg; Washes 1, 3, and 5 with 20 mM imidazole (W1, W2, W3); Elutions 1 and 2 with 250 mM imidazole (E1, E2) = 5 µg. (B) Western blot of 10 µg rEF-Tu probed with pooled sera from BALB/c mice infected i.p. with 10^7^ cfu of *B. thailandensis* (1° Ab, 1∶200; 2° Ab 1∶1000). MW = BenchMark Pre-stained molecular weight ladder. (C) Western blot of 0.5 µg rEF-Tu, 15 µg *B. thailandensis* whole cell lysate (WCL) and 15 µg *B. thailandensis* total membrane protein (TMP) fractions probed with pooled antisera from rEF-Tu-immunized mice (1° Ab, 1∶200; 2° Ab, 1∶1000). MW = SeeBlue Plus2 molecular weight ladder. (D) Western blot of 0.5 µg rEF-Tu, 15 µg *E. coli* WCL and 15 µg *E. coli* TMP fractions probed with monoclonal antibody to *E. coli* β subunit of RNA Polymerase (1° Ab, 1∶1000; 2° Ab, 1∶1000). MW = SeeBlue Plus2 molecular weight ladder.

Prior work suggests that *B. pseudomallei* EF-Tu is present on the bacterial surface and is recognized by convalescent sera from human melioidosis patients [27]. Thus, we hypothesized that EF-Tu may represent a novel immunoprotective antigen. To determine if EF-Tu is recognized during infection in the murine model of melioidosis, we infected a group of BALB/c mice (N = 6) intraperitoneally (i.p.) with 10^7^ cfu of *B. thailandensis* and harvested sera from survivors two weeks later. The pooled sera from infected mice recognized the recombinant, purified preparation of EF-Tu (rEF-Tu) (Figure 2A and B), while sera from uninfected mice did not (not shown). This indicates that EF-Tu is expressed during infection and is recognized by host antibody in the mouse model. Furthermore, these observations indicate that host antibody generated to native EF-Tu during bacterial infection cross-reacts with rEF-Tu. To determine if rEF-Tu could induce antibodies that recognize native EF-Tu, we immunized a group of BALB/c mice (N = 6) subcutaneously with 25 µg rEF-Tu adsorbed to aluminum hydroxide adjuvant and boosted with the same formulation on day 21. On day 35, sera were collected, pooled, and affinity purified for immunoblot of rEF-Tu, as well as whole cell and total membrane protein fractions of *B. thailandensis*. Pooled sera from rEF-Tu-immunized mice recognized the 47 kDa recombinant form of EF-Tu, as well as native EF-Tu in the whole cell lysate and total membrane fraction (Fig. 2C). The bands were excised, digested, and analyzed by MALDI-TOF mass spectrometry to confirm their identity. None of the EF-Tu proteins were detected by Western blot using pooled sera from naïve BALB/c mice (not shown). To rule out cytoplasmic EF-Tu contamination in the membrane fraction, a monoclonal antibody against the β subunit of *E. coli* RNA polymerase (NeoClone) was used to probe *E. coli* cellular fractions prepared in exactly the same manner as *B. thailandensis*. A band at 150 kDa corresponding to the β subunit was observed in the whole cell lysate and was absent in the total membrane preparation (Fig. 2D), indicating that the membrane preparation is free of cytoplasmic contamination.

### Burkholderia EF-Tu is secreted in outer membrane vesicles

**Figure 3:**
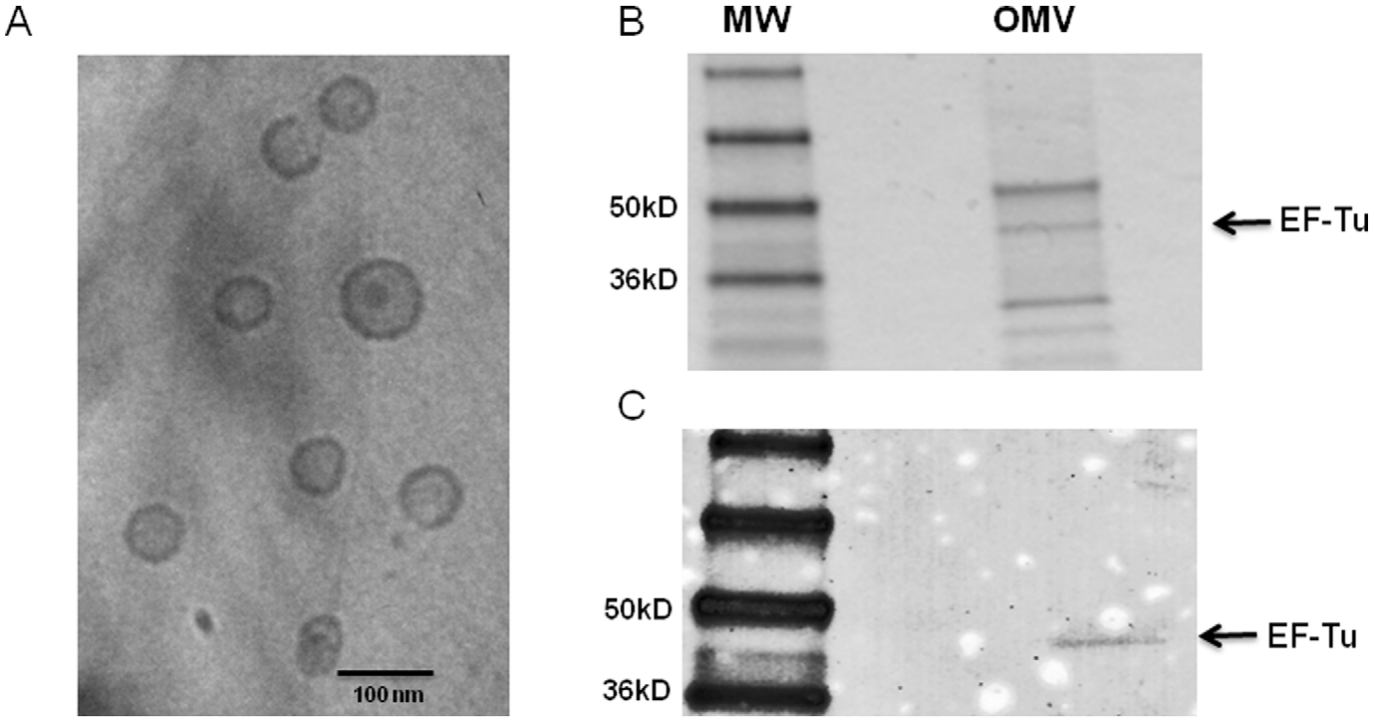
EF-Tu is present in *B. pseudomallei* outer membrane vesicles. (A) Cryo-transmission electron micrograph of purified OMVs prepared from a late logarithmic culture of *B*. *pseuodomallei* strain 1026b. Bar indicates 100 nm. (B) Coomassie-stained gel of OMV preparation (5 µg); MW = SeeBlue plus2 molecular weight ladder. (C) Western blot of OMV preparation using affinity purifed EF-Tu antibody (1∶1000).

EF-Tu has been demonstrated on the surface of several pathogenic bacteria, including *B. pseudomallei* and closely-related *Pseudomonas aeruginosa* [27], [29]. However, we were unsuccessful in our attempts to demonstrate EF-Tu on the surface of *Burkholderia thailandensis* using both immunogold labeling and immunofluoresecnt microscopy. EF-Tu lacks a recognizable signal sequence and the mechanism by which EF-Tu is transported to the bacterial surface has remained an enigma. Recent work with bacterial OMVs has demonstrated that OMVs contain numerous virulence factors, including cytoplasmic, periplasmic, and outer membrane constituents [33]. We therefore considered the possibility that EF-Tu, an abundant bacterial protein, might be shed in OMVs. OMVs were prepared from a late logarithmic culture of *B. pseudomallei* strain 1026b (Figure 3A and 3B) and probed with affinity-purified antibody to EF-Tu. We detected the presence of EF-Tu in *B. pseudomallei* OMVs (Figure 3C), which may partially account for the export of EF-Tu from the bacterial cytoplasm.

### Mucosal and parenteral immunization with EF-Tu yields antigen-specific IgG and IgA

The ability of rEF-Tu to generate antigen-specific IgG that recognizes the native form of EF-Tu indicates its potential use as a vaccine immunogen. We therefore designed a mucosal and parenteral immunization strategy to measure and compare the antibody and CMI responses elicited by rEF-Tu immunization. Groups of BALB/c mice (n = 12) were primed either subcutaneously (s.c.) with 25 µg rEF-Tu adsorbed to aluminum hydroxide or intranasally (i.n.) with 25 µg rEF-Tu and 5 µg CpG ODN 1826. CpG ODN is a well-characterized TLR9 ligand that can be administered parenterally or mucosally to drive type 1 immune responses [34], [35] and can increase vaccine efficacy against *B. pseudomallei* [6], [36], [37]. Adjuvant-only (n = 5) and naïve mice (n = 12) were included as controls. Mice were boosted on day 21 with the same formulations using homologous (s.c. + s.c.; i.n. + i.n.) and heterologous (s.c. + i.n.) prime-boost strategies. Sera and BAL fluid from half (n = 6) of the animals in the immunized and naïve groups were harvested on day 35 and assayed for reactivity with rEF-Tu by ELISA.

**Figure 4:**
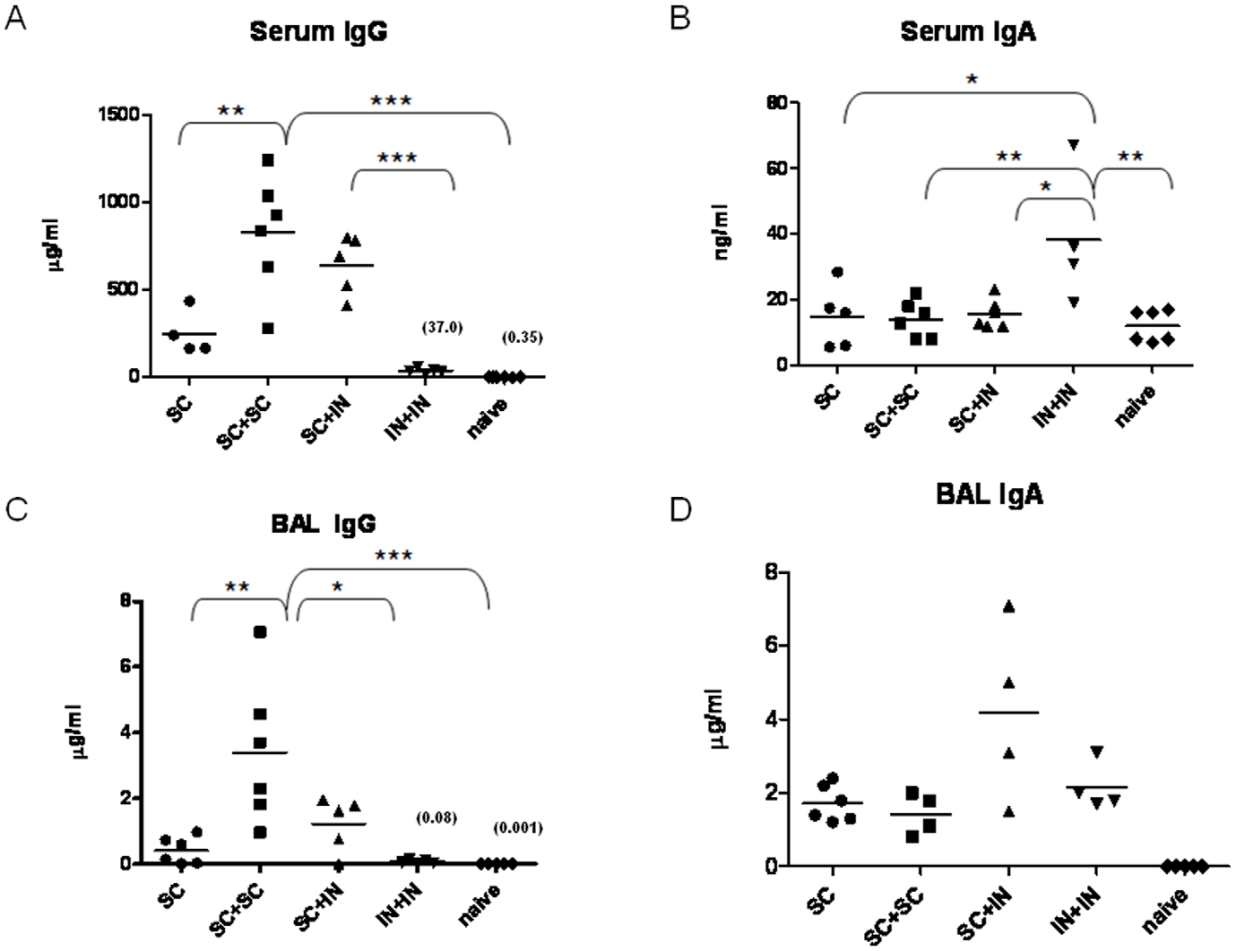
rEF-Tu-specific IgG and IgA concentrations in sera and BAL of immunized mice. Serum IgG (A) and IgA (B) and BAL IgG (C) and IgA (D) were measured by ELISA. SC = subcutaneous immunization with 25 µg rEF-Tu adsorbed 1∶1 with aluminum hydroxide adjuvant. IN = intranasal immunization with 25 µg rEF-Tu admixed with 5 µg CpG adjuvant. Horizontal line represents the median value for each group (N = 6). Median values are provided in parentheses for IN + IN and naïve groups in panels A and C. (*P<0.05, **P<0.01, ***P<0.001 using the Mann-Whitney test).

Antigen-specific serum IgG and IgA concentrations were significantly higher in all immunized groups compared to naïve mouse sera (Fig. 4A and 4B; *P*<0.001). The s.c. + s.c. mice produced the highest concentrations of EF-Tu-specific serum IgG, while the i.n. + i.n. mice produced the lowest concentrations among the immunized groups. In contrast, induction of EF-Tu-specific serum IgA was only observed in the i.n. + i.n. mice (Fig. 4B). Antigen-specific IgG and IgA in the BAL was significantly higher in all immunized groups compared to BAL from naïve mice (*P*<0.001). The s.c. + s.c. group produced the greatest concentrations of EF-Tu-specific BAL IgG (Fig. 4C). EF-Tu-specific IgA was more than 100-fold higher in the BAL than in the serum of immunized animals regardless of the route of immunization. The median concentration of EF-Tu-specific BAL IgA was highest in the s.c. + i.n. group, although it was not statistically different from the other immunized groups (Fig. 4D).

We also assayed IgG1 and IgG2a in the serum and BAL to test for any differences in the type 1 and type 2 immune responses elicited in each group. Mice immunized s.c. + s.c. demonstrated IgG1:IgG2a ratios of 5.6 and 140 in the sera and BAL, respectively (Table 2). The predominance of IgG1 is more characteristic of a type 2 immune response [38]. Mice immunized s.c. + i.n. and i.n. + i.n. displayed serum IgG1:IgG2a ratios of 1.5 and 0.004, respectively, and demonstrated a shift from IgG1 to IgG2a in the BAL as well (Table 2). These results indicate the generation of a stronger type 1 immune response in the mucosally immunized groups versus those immunized parenterally.

**Table 2:** Serum and BAL EF-Tu specific IgG1 and IgG2a concentrations (µg/ml) and ratios.

	Serum			BAL		
**Group**	**IgG1**	**IgG2a**	**ratio**	**IgG1**	**IgG2a**	**ratio**
**SC**	124.5	13.7	9.1	0.47	0.22	22
**SC+SC**	333.7	59.6	5.6	3.7	0.02	140
**SC+IN**	93.0	63.2	1.5	1.5	0.04	31
**IN+IN**	0.28	63.3	0.004	0.07	0.009	8.4

IgG1 and IgG2a were measured by ELISA using sera and BAL from immunized mice (N = 6). IgG1:IgG2a ratios >1 indicate a type 2 humoral immune response, while ratios <1 indicate a type 1 cellular immune response.

### Th1 and Th2 cytokine responses in EF-Tu restimulated splenoctyes

**Figure 5:**
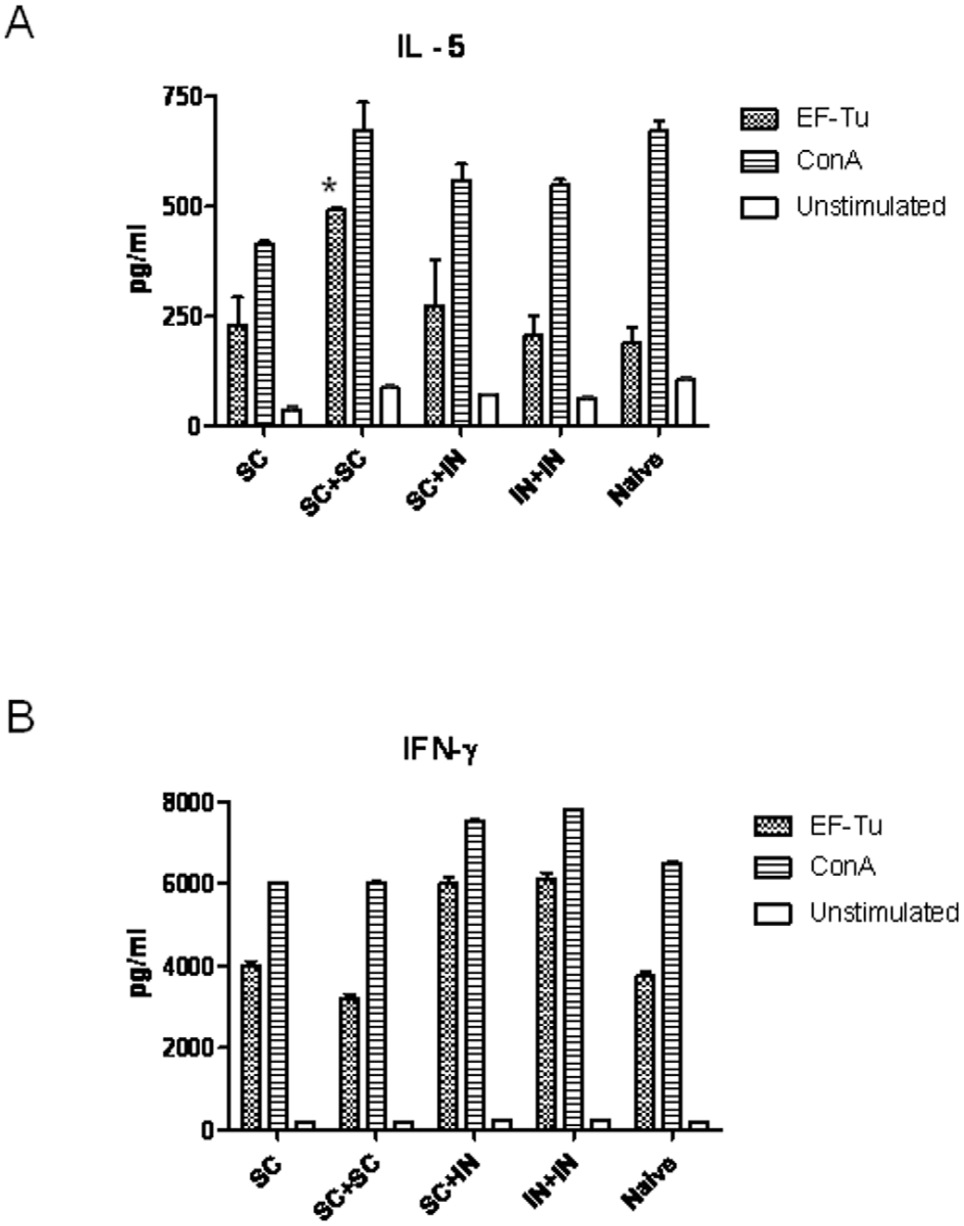
Th1 and Th2 cytokine responses to rEF-Tu in restimulated splenocytes from immunized mice. Splenocytes from individual mice in each treatment group (N = 6) were restimulated in triplicate with rEF-Tu (1 µg) or ConA (1 µg) or left unstimulated, and cell culture supernatants were assayed in duplicate on day 3 for IL-5 (A) and IFN-γ (B) cytokine production using a multiplex assay. Error bars represent the standard error of the mean (SEM) for each group (*P<0.05 using a two-way ANOVA).

A Th1-driven CMI response, in concert with the production of specific antibodies, is likely essential for vaccine efficacy against *B. pseudomallei* [9], [16]. To assess antigen-specific T cell responses in rEF-Tu immunized mice, spleens were harvested on day 35 (2 weeks post-immunization) and restimulated *in vitro* with rEF-Tu. Cell culture supernatants were assayed on day three for IFN-γ and IL-5 production as an indication of Th1 and Th2 responses, respectively. Mice that were immunized s.c + s.c. produced significantly higher levels of IL-5 compared to naïve animals (Fig. 5A; *P*<0.05) upon restimulation with rEF-Tu. In contrast, mice that received one dose of rEF-Tu s.c. and both mouse groups boosted mucosally (s.c. + i.n. and i.n. + i.n.) produced similar levels of IL-5 compared to naïve mice (Fig. 5A). Both groups that were boosted mucosally (s.c. + i.n. and i.n. + i.n.) produced higher levels of IFN-γ than mice that were immunized parenterally (s.c. only and s.c. + s.c.) and naïve mice (Fig. 5B), although this increase was not statistically significant.

### Mucosal immunization with EF-Tu reduces bacterial burden in the lung

**Figure 6:**
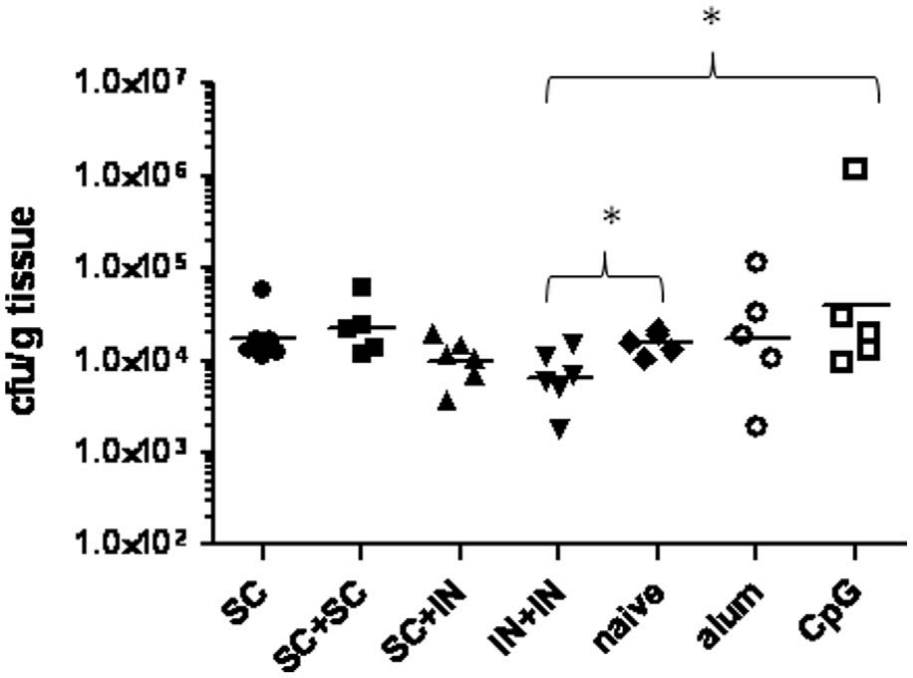
Bacterial burden in lungs of EF-Tu immunized and challenged mice. Lung bacterial burdens (cfu/g tissue) were determined in naïve (N = 6), adjuvant-only (N = 5), and immunized (N = 6) mice 24 hrs post-aerosol challenge with 5×10^5^ cfu (∼LD_50_) of *B. thailandensis*. SC = subcutaneously immunized; IN = intranasally immunized. Horizontal line represents the geometric mean for each group. (*P<0.05 using the Mann-Whitney test).

We challenged EF-Tu-immunized mice with *B. thailandensis* as a preliminary measure of protective capacity in an *in vivo* test system. *B. thailandensis* is not considered a human pathogen, however it is lethal in inbred mouse strains (BALB/c and C57Bl/6) at aerosol challenge doses of 1×10^5^ cfu or higher [20], [21]. We therefore challenged mice (N = 5–6) in immunized, adjuvant-only, and naïve groups with 5×10^5^ cfu (∼LD_50_) of *B. thailandensis* by aerosol on day 35. All mice were sacrificed 24 hr later to assess lung bacterial burdens since there is a direct correlation between lung bacterial burden and disease progression in this acute pneumonia model [20], [21]. Mice that were primed s.c. and boosted either s.c. or i.n. (s.c. + s.c., s.c. + i.n.) had similar numbers of bacteria in the lungs compared to control mice (Fig. 6). Significantly lower bacterial burdens in lung tissues were observed in the i.n. + i.n. mice when compared to the adjuvant only (CpG) and naïve groups (*P*<0.05; Fig. 6).

## Discussion

There is currently no effective vaccine against *B. pseudomallei*, and traditional vaccine attempts have been largely ineffective at preventing the inhalational form of disease in animal models [14]. Therefore, alternative vaccination strategies that incorporate recent advances in adjuvant biology and mucosal immunology deserve investigation. Our approach employed immunoproteomics to identify proteins that could be utilized as subunit vaccine antigens and delivered mucosally. Of the 11 proteins that we identified, three (EF-Tu, AhpC, and DnaK) were previously recognized by Harding *et al*. [27] using a similar approach with convalescent sera from melioidosis patients. The co-recognition of these particular *B. pseudomallei* antigens by two independent laboratories reinforces their potential value as vaccine immunogens. We therefore selected one of the three, EF-Tu, as our first test antigen since both AhpC and DnaK have received considerable attention elsewhere for related bio-threat agents [39]–[42].

Its traditional cytoplasmic role in protein synthesis would render EF-Tu an unlikely candidate for a protective subunit vaccine. However, EF-Tu is one of the most abundant and conserved bacterial proteins (100% amino acid identity among *B. thailandensis*, *B*. *mallei*, and five different strains of *B. pseudomallei* – Table 1 and Files S1 and S2) and is a major component of the bacterial membrane cytoskeleton [43], [44]. EF-Tu comprises as much as 5–10% of the cytoplasmic protein in all bacteria investigated, and it may be functionally analogous to actin as it can polymerize into bundle filaments and bind DNase1[43], [45]. Emerging evidence demonstrates that EF-Tu may play a previously under-appreciated role as a bacterial virulence factor. For example, surface-translocated EF-Tu mediates binding to fibronectin and other host proteins for *Mycoplasma pneumoniae* and *Pseudomonas aeruginosa*, and EF-Tu can facilitate invasion of host cells by *Francisella tularensis* via interaction with nucleolin [29], [30], [32]. Furthermore, immunoproteomic-based approaches for antigen discovery against other intracellular bacterial pathogens have identified EF-Tu as an immunodominant protein [46], [47]. Taken together, these studies lend support to our observations of immunogenic EF-Tu in the membrane of *B. thailandensis* and that reported elsewhere for *B. pseudomallei* [27]. Although we were unable to demonstrate EF-Tu on the surface of *B. thailandensis*, we did observe EF-Tu in the OMVs shed from *B. pseudomallei* during *in vitro* growth. This may partially account for the generation of host antibody against EF-Tu since OMVs have been observed to activate B cells [33]. In this study, active immunization of mice with EF-Tu generated high concentrations of antigen-specific IgG that recognized both the recombinant and native forms of EF-Tu. As far as we know, this work is the first application and evaluation of EF-Tu as a vaccine immunogen for a bacterial pathogen. Like the bacterial antigens, flagellin and LPS (both highly-evaluated as vaccine constituents), EF-Tu is abundantly present and highly immunogenic during *B*. *pseudomallei* infection in humans [27] and animal models of melioidosis and thus deserves investigation. Furthermore, bacterial EF-Tu and human EF2 share only 17% identity at the amino acid level and are not functionally interchangeable (File S1) [44]. We also observed no cross-reactivity of EF-Tu-specific antibody with mammalian tissue by Western blot (File S3). Thus, the potential for bacterial EF-Tu to induce autoimmune disease in vaccinated individuals appears negligible.

Our heterologous and homologous prime/boost immunization studies compared the traditional parenteral route of immunization with aluminum hydroxide as the adjuvant to an intranasal formulation of rEF-Tu admixed with CpG ODN, an adjuvant capable of polarizing the immune response to Th1 and enhancing mucosal IgA, systemic antibody, and T cell immunity [34], [35]. It has been proposed that *B. pseudomallei* may utilize the NALT as a portal of entry in murine melioidosis [17]. Therefore, the i.n. route of immunization may better prevent mucosal infections through the priming and activation of local antimicrobial immunity. To test this hypothesis, we challenged both parenterally and mucosally immunized mice with 5×10^5^ cfu of *B. thailandensis* using a nose-only inhalation exposure chamber. We and others have previously demonstrated that aerosol infection of BALB/c mice with *B. thailandensis* is an excellent surrogate model for the acute pneumonic form of disease caused by *B. pseudomallei* and is capable of reproducing the major lung pathology of murine melioidosis [20], [21]. Furthermore, there is a direct correlation between lung bacterial burden and disease progression in the murine model [20], [21]. The reduced bacterial numbers observed *only* in the lungs of mice that were immunized mucosally with EF-Tu/CpG suggests that EF-Tu immunization may influence protection and that the route of immunization may be critical. Moreover, the early reduction in bacterial burden in the i.n. + i.n. group cannot exclusively be attributed to the immunoprotective capacity of CpG [48] because mice immunized i.n. with CpG ODN 1826 alone had similar, if not slightly higher, numbers of bacteria compared to naïve mice that were challenged (Fig. 6).

Past vaccine attempts against *B. pseudomallei* failed to confer complete protection despite the induction of a robust antibody response; however, humoral immunity will likely be an essential component of any vaccine against this organism [16]. Previous work demonstrated a 0% survival rate in mice immunized with *B. pseudomallei*-pulsed dendritic cells though the immunization generated a substantial cell-mediated immune response [16]. Protection could be achieved when the mice were boosted with heat-killed bacteria and correlated with the production of high *B. pseudomallei*-specific antibody titers [16]. Immunization with EF-Tu yielded high concentrations of antigen-specific IgG in the sera and BAL by both the parenteral and mucosal immunization regimens. However, EF-Tu-specific IgG levels did not correlate with the observed differences in lung bacterial burdens in immunized mice in our study. In addition to IgG, secretory IgA may play a role in protection against inhalational pathogens as previously demonstrated for *Bordetella pertussis* [49]. Undetectable to very low levels of EF-Tu-specific IgA were observed in the sera of immunized mice regardless of the route of immunization. In contrast, EF-Tu-specific IgA was significantly elevated in the BAL of immunized mice compared to naïve mice, but there was no statistical difference among any of the immunized groups. Therefore, IgA concentrations also may not account for the differences observed in lung bacterial burdens at the time point examined.

Although antibodies contribute to protection against *B. pseudomallei* [16] a robust CMI response is likely required for ultimate clearance of internalized bacteria [16], [50]. Antigen-specific T cells, particularly CD4^+^ T cells, are important sources of IFN-γ and are essential for host resistance to acute and chronic infection with *B. pseudomallei* [50]. EF-Tu was recently shown to elicit memory CD4^+^ T cells in cattle immunized with outer membrane protein preparations of the rickettsial pathogen, *Anaplasma marginale* [51]. Our work corroborates their findings as we demonstrated both Th1 (IFN-γ) and Th2 (IL-5) cytokine production in EF-Tu-restimulated splenocytes that reflected both the adjuvant used and the route of immunization. In other words, the parenteral immunization strategy that incorporated aluminum hydroxide as adjuvant promoted Th2 responses to rEF-Tu, while the mucosal administration of rEF-Tu with CpG polarized the immune response towards Th1. This is also supported by the IgG1:IgG2a ratios in the sera and BAL that demonstrated a Th1 polarization in mucosally immunized mice (Table 2). These results are not entirely unexpected, and it is plausible that the antigen-specific Th1 response elicited by mucosal immunization with rEF-Tu/CpG is responsible for the reduced bacterial burden observed early in the lungs of the i.n. + i.n. group. Considering the predominance of EF-Tu in the bacterial cell, further analysis of EF-Tu-specific CD4^+^ T memory cells is clearly warranted.

The lack of adequate treatment and prevention against melioidosis necessitates the development of a vaccine against *B. pseudomallei* [14]. Inhalation of *B. pseudomallei* is a natural route of infection, and it represents the primary route of exposure in a deliberate biological attack. A *B. pseudomallei* vaccine should therefore be efficacious against this route of infection. We identified EF-Tu, a protein best recognized for its role in bacterial protein synthesis, as a subunit vaccine candidate against pathogenic *Burkholderia*. Our data indicate that recombinant EF-Tu is immunogenic, inducing antigen-specific antibody and CMI responses. To our knowledge, this is the first *in vivo* demonstration of the utility of bacterial EF-Tu as a vaccine immunogen. Mucosal immunization with EF-Tu/CpG reduced lung bacterial loads in mice challenged with a lethal dose of *B. thailandensis* by aerosol. Further immunization and challenge studies with virulent *B. pseudomallei* will examine the protective efficacy of EF-Tu and represent the next steps in evaluation of EF-Tu as a viable vaccine candidate.

## Supporting Information

File S1 Elongation factor Tu amino acid alignment and percent identity for Burkholderia thailandensis, B. pseudomallei, B. mallei, E. coli, and Homo sapiens.
(0.03 MB DOCX)

File S2 Elongation factor Tu amino acid alignment and percent identity among five sequenced isolates of B. pseudomallei.
(0.03 MB DOCX)

File S3 Antibody against bacterial EF-Tu does not react with mammalian tissue. (A) Coomassie stained gel of mouse lung (Lu), liver (Lv), and spleen (S) homogenates. MW = SeeBlue plus2 molecular weight ladder. (B) Western blot of mouse tissues using 1∶100 dilution of affinity purified EF-Tu IgG.
(0.24 MB TIF)

## References

[1] Cheng AC, Currie BJ (2005). Melioidosis: epidemiology, pathophysiology, and management.. Clin Microbiol Rev.

[2] Wiersinga WJ, van der Poll T (2009). Immunity to Burkholderia pseudomallei.. Curr Opin Infect Dis.

[3] Leelarasamee A (2004). Recent development in melioidosis.. Curr Opin Infect Dis.

[4] Peacock SJ (2006). Melioidosis.. Curr Opin Infect Dis.

[5] Jones AL, Beveridge TJ, Woods DE (1996). Intracellular survival of Burkholderia pseudomallei.. Infect Immun.

[6] Harland DN, Chu K, Haque A, Nelson M, Walker NJ (2007). Identification of a LolC homologue in Burkholderia pseudomallei, a novel protective antigen for melioidosis.. Infect Immun.

[7] Jones SM, Ellis JF, Russell P, Griffin KF, Oyston PC (2002). Passive protection against Burkholderia pseudomallei infection in mice by monoclonal antibodies against capsular polysaccharide, lipopolysaccharide or proteins.. J Med Microbiol.

[8] Nelson M, Prior JL, Lever MS, Jones HE, Atkins TP (2004). Evaluation of lipopolysaccharide and capsular polysaccharide as subunit vaccines against experimental melioidosis.. J Med Microbiol.

[9] Haque A, Chu K, Easton A, Stevens MP, Galyov EE (2006). A live experimental vaccine against Burkholderia pseudomallei elicits CD4+ T cell-mediated immunity, priming T cells specific for 2 type III secretion system proteins.. J Infect Dis.

[10] Hara Y, Mohamed R, Nathan S (2009). Immunogenic Burkholderia pseudomallei outer membrane proteins as potential candidate vaccine targets.. PLoS One.

[11] Druar C, Yu F, Barnes JL, Okinaka RT, Chantratita N (2008). Evaluating Burkholderia pseudomallei Bip proteins as vaccines and Bip antibodies as detection agents.. FEMS Immunol Med Microbiol.

[12] Breitbach K, Kohler J, Steinmetz I (2008). Induction of protective immunity against Burkholderia pseudomallei using attenuated mutants with defects in the intracellular life cycle.. Trans R Soc Trop Med Hyg.

[13] Stevens MP, Haque A, Atkins T, Hill J, Wood MW (2004). Attenuated virulence and protective efficacy of a Burkholderia pseudomallei bsa type III secretion mutant in murine models of melioidosis.. Microbiology.

[14] Bondi SK, Goldberg JB (2008). Strategies toward vaccines against Burkholderia mallei and Burkholderia pseudomallei.. Expert Rev Vaccines.

[15] Newman M, Newman M (1995). Immunological and Formulation Design Considerations for Subunit Vaccines;.

[16] Healey GD, Elvin SJ, Morton M, Williamson ED (2005). Humoral and cell-mediated adaptive immune responses are required for protection against Burkholderia pseudomallei challenge and bacterial clearance postinfection.. Infect Immun.

[17] Owen SJ, Batzloff M, Chehrehasa F, Meedeniya A, Casart Y (2009). Nasal-Associated Lymphoid Tissue and Olfactory Epithelium as Portals of Entry for Burkholderia pseudomallei in Murine Melioidosis.. J Infect Dis.

[18] Stevens JM, Ulrich RL, Taylor LA, Wood MW, Deshazer D (2005). Actin-binding proteins from Burkholderia mallei and Burkholderia thailandensis can functionally compensate for the actin-based motility defect of a Burkholderia pseudomallei bimA mutant.. J Bacteriol.

[19] Kim HS, Schell MA, Yu Y, Ulrich RL, Sarria SH (2005). Bacterial genome adaptation to niches: divergence of the potential virulence genes in three Burkholderia species of different survival strategies.. BMC Genomics.

[20] West TE, Frevert CW, Liggitt HD, Skerrett SJ (2008). Inhalation of Burkholderia thailandensis results in lethal necrotizing pneumonia in mice: a surrogate model for pneumonic melioidosis.. Trans R Soc Trop Med Hyg.

[21] Morici LA, Heang J, Tate T, Didier PJ, Roy CJ Differential susceptibility of inbred mouse strains to Burkholderia thailandensis aerosol infection.. Microb Pathog.

[22] Wiersinga WJ, de Vos AF, de Beer R, Wieland CW, Roelofs JJ (2008). Inflammation patterns induced by different Burkholderia species in mice.. Cell Microbiol.

[23] Moe GR, Zuno-Mitchell P, Hammond SN, Granoff DM (2002). Sequential immunization with vesicles prepared from heterologous Neisseria meningitidis strains elicits broadly protective serum antibodies to group B strains.. Infect Immun.

[24] Bauman SJ, Kuehn MJ (2006). Purification of outer membrane vesicles from Pseudomonas aeruginosa and their activation of an IL-8 response.. Microbes Infect.

[25] Glynn A, Freytag LC, Clements JD (2005). Effect of homologous and heterologous prime-boost on the immune response to recombinant plague antigens.. Vaccine.

[26] Rappuoli R (2000). Reverse vaccinology.. Curr Opin Microbiol.

[27] Harding SV, Sarkar-Tyson M, Smither SJ, Atkins TP, Oyston PC (2007). The identification of surface proteins of Burkholderia pseudomallei.. Vaccine.

[28] Yokosawa H, Inoue-Yokosawa N, Arai KI, Kawakita M, Kaziro Y (1973). The role of guanosine triphosphate hydrolysis in elongation factor Tu-promoted binding of aminoacyl transfer ribonucleic acid to ribosomes.. J Biol Chem.

[29] Kunert A, Losse J, Gruszin C, Huhn M, Kaendler K (2007). Immune evasion of the human pathogen Pseudomonas aeruginosa: elongation factor Tuf is a factor H and plasminogen binding protein.. J Immunol.

[30] Balasubramanian S, Kannan TR, Baseman JB (2008). The surface-exposed carboxyl region of Mycoplasma pneumoniae elongation factor Tu interacts with fibronectin.. Infect Immun.

[31] Balasubramanian S, Kannan TR, Hart PJ, Baseman JB (2009). Amino acid changes in elongation factor Tu of Mycoplasma pneumoniae and Mycoplasma genitalium influence fibronectin binding.. Infect Immun.

[32] Barel M, Hovanessian AG, Meibom K, Briand JP, Dupuis M (2008). A novel receptor - ligand pathway for entry of Francisella tularensis in monocyte-like THP-1 cells: interaction between surface nucleolin and bacterial elongation factor Tu.. BMC Microbiol.

[33] Amano A, Takeuchi H, Furuta N Outer membrane vesicles function as offensive weapons in host-parasite interactions.. Microbes Infect.

[34] Freytag LC, Clements JD (2005). Mucosal adjuvants.. Vaccine.

[35] Klinman DM, Klaschik S, Sato T, Tross D (2009). CpG oligonucleotides as adjuvants for vaccines targeting infectious diseases.. Adv Drug Deliv Rev.

[36] Chen YS, Hsiao YS, Lin HH, Liu Y, Chen YL (2006). CpG-modified plasmid DNA encoding flagellin improves immunogenicity and provides protection against Burkholderia pseudomallei infection in BALB/c mice.. Infect Immun.

[37] Elvin SJ, Healey GD, Westwood A, Knight SC, Eyles JE (2006). Protection against heterologous Burkholderia pseudomallei strains by dendritic cell immunization.. Infect Immun.

[38] DuBois AB, Freytag LC, Clements JD (2007). Evaluation of combinatorial vaccines against anthrax and plague in a murine model.. Vaccine.

[39] Ariel N, Zvi A, Makarova KS, Chitlaru T, Elhanany E (2003). Genome-based bioinformatic selection of chromosomal Bacillus anthracis putative vaccine candidates coupled with proteomic identification of surface-associated antigens.. Infect Immun.

[40] Gat O, Grosfeld H, Ariel N, Inbar I, Zaide G (2006). Search for Bacillus anthracis potential vaccine candidates by a functional genomic-serologic screen.. Infect Immun.

[41] Savitt AG, Mena-Taboada P, Monsalve G, Benach JL (2009). Francisella tularensis infection-derived monoclonal antibodies provide detection, protection, and therapy.. Clin Vaccine Immunol.

[42] Delpino MV, Estein SM, Fossati CA, Baldi PC, Cassataro J (2007). Vaccination with Brucella recombinant DnaK and SurA proteins induces protection against Brucella abortus infection in BALB/c mice.. Vaccine.

[43] Mayer F (2003). Cytoskeletons in prokaryotes.. Cell Biol Int.

[44] Jonak J (2007). Bacterial elongation factors EF-Tu, their mutants, chimeric forms, and domains: isolation and purification.. J Chromatogr B Analyt Technol Biomed Life Sci.

[45] Beck BD, Arscott PG, Jacobson A (1978). Novel properties of bacterial elongation factor Tu.. Proc Natl Acad Sci U S A.

[46] Bunk S, Susnea I, Rupp J, Summersgill JT, Maass M (2008). Immunoproteomic identification and serological responses to novel Chlamydia pneumoniae antigens that are associated with persistent C. pneumoniae infections.. J Immunol.

[47] Gupta MK, Subramanian V, Yadav JS (2009). Immunoproteomic identification of secretory and subcellular protein antigens and functional evaluation of the secretome fraction of Mycobacterium immunogenum, a newly recognized species of the Mycobacterium chelonae-Mycobacterium abscessus group.. J Proteome Res.

[48] Wongratanacheewin S, Kespichayawattana W, Intachote P, Pichyangkul S, Sermswan RW (2004). Immunostimulatory CpG oligodeoxynucleotide confers protection in a murine model of infection with Burkholderia pseudomallei.. Infect Immun.

[49] Watanabe M, Nagai M (2003). Role of systemic and mucosal immune responses in reciprocal protection against Bordetella pertussis and Bordetella parapertussis in a murine model of respiratory infection.. Infect Immun.

[50] Haque A, Easton A, Smith D, O'Garra A, Van Rooijen N (2006). Role of T cells in innate and adaptive immunity against murine Burkholderia pseudomallei infection.. J Infect Dis.

[51] Lopez JE, Beare PA, Heinzen RA, Norimine J, Lahmers KK (2008). High-throughput identification of T-lymphocyte antigens from Anaplasma marginale expressed using in vitro transcription and translation.. J Immunol Methods.

