# Supplementary material for: Immunospecific Responses to Bacterial Elongation Factor Tu during Burkholderia Infection and Immunization
Source: PLoS One. 2010 Dec 17;5(12):e14361. doi: 10.1371/journal.pone.0014361 (PMC3003680; doi:10.1371/journal.pone.0014361)
Supplement: File S1 — Elongation factor Tu amino acid alignment and percent identity for Burkholderia thailandensis, B. pseudomallei, B. mallei, E. coli, and Homo sapiens. (0.03 MB DOCX) [file pone.0014361.s001.docx]

**elongation factor Tu [Burkholderia thailandensis E264]**

NCBI Reference Sequence: YP_443574.1

>gi|83721154|ref|YP_443574.1| elongation factor Tu [Burkholderia thailandensis E264]

MAKEKFERTKPHVNVGTIGHVDHGKTTLTAAIATVLSAKFGGEAKKYDEIDAAPEEKARGITINTAHIEY

ETANRHYAHVDCPGHADYVKNMITGAAQMDGAILVCSAADGPMPQTREHILLARQVGVPYIIVFLNKCDM

VDDAELLELVEMEVRELLSKYDFPGDDTPIIKGSAKLALEGDKGELGEVAIMNLADALDTYIPTPERAVD

GAFLMPVEDVFSISGRGTVVTGRVERGVIKVGEEIEIVGIKATAKTTCTGVEMFRKLLDQGQAGDNVGIL

LRGTKREDVERGQVLAKPGSITPHTHFTAEVYVLSKDEGGRHTPFFNNYRPQFYFRTTDVTGSIELPKDK

EMVMPGDNVSITVKLIAPIAMEEGLRFAIREGGRTVGAGVVAKIIE

Uniprot/Swiss prot # Q2SU25

**elongation factor Tu [Burkholderia pseudomallei K96243]**

GenBank: CAH37239.1

>gi|52211250|emb|CAH37239.1| elongation factor Tu [Burkholderia pseudomallei K96243]

MAKEKFERTKPHVNVGTIGHVDHGKTTLTAAIATVLSAKFGGEAKKYDEIDAAPEEKARGITINTAHIEY

ETANRHYAHVDCPGHADYVKNMITGAAQMDGAILVCSAADGPMPQTREHILLARQVGVPYIIVFLNKCDM

VDDAELLELVEMEVRELLSKYDFPGDDTPIIKGSAKLALEGDKGELGEVAIMNLADALDTYIPTPERAVD

GAFLMPVEDVFSISGRGTVVTGRVERGVIKVGEEIEIVGIKATAKTTCTGVEMFRKLLDQGQAGDNVGIL

LRGTKREDVERGQVLAKPGSITPHTHFTAEVYVLSKDEGGRHTPFFNNYRPQFYFRTTDVTGSIELPKDK

EMVMPGDNVSITVKLIAPIAMEEGLRFAIREGGRTVGAGVVAKIIE

Uniprot/Swiss prot # Q63PZ6

**elongation factor Tu [Burkholderia mallei ATCC 23344]**

NCBI Reference Sequence: YP_104168.1

>gi|53723856|ref|YP_104168.1| elongation factor Tu [Burkholderia mallei ATCC 23344]

MAKEKFERTKPHVNVGTIGHVDHGKTTLTAAIATVLSAKFGGEAKKYDEIDAAPEEKARGITINTAHIEY

ETANRHYAHVDCPGHADYVKNMITGAAQMDGAILVCSAADGPMPQTREHILLARQVGVPYIIVFLNKCDM

VDDAELLELVEMEVRELLSKYDFPGDDTPIIKGSAKLALEGDKGELGEVAIMNLADALDTYIPTPERAVD

GAFLMPVEDVFSISGRGTVVTGRVERGVIKVGEEIEIVGIKATAKTTCTGVEMFRKLLDQGQAGDNVGIL

LRGTKREDVERGQVLAKPGSITPHTHFTAEVYVLSKDEGGRHTPFFNNYRPQFYFRTTDVTGSIELPKDK

EMVMPGDNVSITVKLIAPIAMEEGLRFAIREGGRTVGAGVVAKIIE

Uniprot/Swiss prot # Q626K3

**protein chain elongation factor EF-Tu [Escherichia coli str. K-12 substr. MG1655]**

NCBI Reference Sequence: NP_418407.1

>gi|16131810|ref|NP_418407.1| protein chain elongation factor EF-Tu (duplicate of tufA) [Escherichia coli str. K-12 substr. MG1655]

MSKEKFERTKPHVNVGTIGHVDHGKTTLTAAITTVLAKTYGGAARAFDQIDNAPEEKARGITINTSHVEY

DTPTRHYAHVDCPGHADYVKNMITGAAQMDGAILVVAATDGPMPQTREHILLGRQVGVPYIIVFLNKCDM

VDDEELLELVEMEVRELLSQYDFPGDDTPIVRGSALKALEGDAEWEAKILELAGFLDSYIPEPERAIDKP

FLLPIEDVFSISGRGTVVTGRVERGIIKVGEEVEIVGIKETQKSTCTGVEMFRKLLDEGRAGENVGVLLR

GIKREEIERGQVLAKPGTIKPHTKFESEVYILSKDEGGRHTPFFKGYRPQFYFRTTDVTGTIELPEGVEM

VMPGDNIKMVVTLIHPIAMDDGLRFAIREGGRTVGAGVVAKVLS

Uniprot/Swiss prot # P0CE48

# human elongation factor 2 [Homo sapiens]

GenBank: CAA77750.1

>gi|31108|emb|CAA77750.1| human elongation factor 2 [Homo sapiens]

MVNFTVDQIRAIMDKKANIRNMSVIAHVDHGKSTLTDSLVCKAGIIASARAGETRFTDTRKDEQERCITI

KSTAISLFYELSENDLNFIKQSKDGAGFLINLIDSPGHVDFSSEVTAALRVTDGALVVVDCVSGVCVQTE

TVLRQAIAERIKPVLMMNKMDRALLELQLEPEELYQTFQRIVENVNVIISTYGEGESGPMGNIMIDPVLG

TVGFGSGLHGWAFTLKQFAEMYVAKFAAKGEGQLGPAERAKKVEDMMKKLWGDRYFDPANGKFSKSATSP

EGKKLPRTFCQLILDPIFKVFDAIMNFKKEETAKLIEKLDIKLDSEDKDKEGKPLLKAVMRRWLPAGDAL

LQMITIHLPSPVTAQKYRCELLYEGPPDDEAAMGIKSCDPKGPLMMYISKMVPTSDKGRFYAFGRVFSGL

VSTGLKVRIMGPNYTPGKKEDLYLKPIQRTILMMGRYVEPIEDVPCGNIVGLVGVDQFLVKTGTITTFEH

AHNMRVMKFSVSPVVRVAVEAKNPADLPKLVEGLKRLAKSDPMVQCIIEESGEHIIAGAGELHLEICLKD

LEEDHACIPIKKSDPVVSYRETVSEESNVLCLSKSPNKHNRLYMKARPFPDGLAEDIDKGEVSARQELKQ

RARYLAEKYEWDVAEARKIWCFGPDGTGPNILTDITKGVQYLNEIKDSVVAGFQWATKEGALCEENMRGV

RFDVHDVTLHADAIHRGGGQIIPTARRCLYASVLTAQPRLMEPIYLVEIQCPEQVVGGIYGVLNRKRGHV

FEESQVAGTPMFVVKAYLPVNESFGFTADLRSNTGGQAFPQCVFDHWQILPGDPFDNSSRPSQVVAETRK

RKGLKEGIPALDNFLDKL

CLUSTAL 2.0.12 multiple sequence alignment

gi|83721154|ref|YP_443574.1| -------MAKEKFERTKPHVNVGTIGHVDHGKTTLTAAIATVLSAKFGGE 43

gi|52211250|emb|CAH37239.1| -------MAKEKFERTKPHVNVGTIGHVDHGKTTLTAAIATVLSAKFGGE 43

gi|53723856|ref|YP_104168.1| -------MAKEKFERTKPHVNVGTIGHVDHGKTTLTAAIATVLSAKFGGE 43

gi|16131810|ref|NP_418407.1| -------MSKEKFERTKPHVNVGTIGHVDHGKTTLTAAITTVLAKTYGGA 43

gi|31108|emb|CAA77750.1| MVNFTVDQIRAIMDKKANIRNMSVIAHVDHGKSTLTDSLVCKAGIIASAR 50

: :::. *:..*.******:*** ::. . ..

gi|83721154|ref|YP_443574.1| AKKYDEIDAAPEEKARGITIN--TAHIEYETANRHY-------------- 77

gi|52211250|emb|CAH37239.1| AKKYDEIDAAPEEKARGITIN--TAHIEYETANRHY-------------- 77

gi|53723856|ref|YP_104168.1| AKKYDEIDAAPEEKARGITIN--TAHIEYETANRHY-------------- 77

gi|16131810|ref|NP_418407.1| ARAFDQIDNAPEEKARGITIN--TSHVEYDTPTRHY-------------- 77

gi|31108|emb|CAA77750.1| AGETRFTDTRKDEQERCITIKSTAISLFYELSENDLNFIKQSKDGAGFLI 100

* * :*: * ***: : : *: . ..

gi|83721154|ref|YP_443574.1| AHVDCPGHADYVKNMITGAAQMDGAILVCSAADGPMPQTR---------- 117

gi|52211250|emb|CAH37239.1| AHVDCPGHADYVKNMITGAAQMDGAILVCSAADGPMPQTR---------- 117

gi|53723856|ref|YP_104168.1| AHVDCPGHADYVKNMITGAAQMDGAILVCSAADGPMPQTR---------- 117

gi|16131810|ref|NP_418407.1| AHVDCPGHADYVKNMITGAAQMDGAILVVAATDGPMPQTR---------- 117

gi|31108|emb|CAA77750.1| NLIDSPGHVDFSSEVTAALRVTDGALVVVDCVSGVCVQTETVLRQAIAER 150

:*.***.*: .:: :. ***::* ...* **.

gi|83721154|ref|YP_443574.1| --------------------------------------------------

gi|52211250|emb|CAH37239.1| --------------------------------------------------

gi|53723856|ref|YP_104168.1| --------------------------------------------------

gi|16131810|ref|NP_418407.1| --------------------------------------------------

gi|31108|emb|CAA77750.1| IKPVLMMNKMDRALLELQLEPEELYQTFQRIVENVNVIISTYGEGESGPM 200

gi|83721154|ref|YP_443574.1| EHILLARQVGVP-------------------YIIVFLNK----------- 137

gi|52211250|emb|CAH37239.1| EHILLARQVGVP-------------------YIIVFLNK----------- 137

gi|53723856|ref|YP_104168.1| EHILLARQVGVP-------------------YIIVFLNK----------- 137

gi|16131810|ref|NP_418407.1| EHILLGRQVGVP-------------------YIIVFLNK----------- 137

gi|31108|emb|CAA77750.1| GNIMIDPVLGTVGFGSGLHGWAFTLKQFAEMYVAKFAAKGEGQLGPAERA 250

:*:: :*. *: * *

gi|83721154|ref|YP_443574.1| ---------------------------------------CDMVDD----- 143

gi|52211250|emb|CAH37239.1| ---------------------------------------CDMVDD----- 143

gi|53723856|ref|YP_104168.1| ---------------------------------------CDMVDD----- 143

gi|16131810|ref|NP_418407.1| ---------------------------------------CDMVDD----- 143

gi|31108|emb|CAA77750.1| KKVEDMMKKLWGDRYFDPANGKFSKSATSPEGKKLPRTFCQLILDPIFKV 300

*::: *

gi|83721154|ref|YP_443574.1| -AELLELVEMEVRELLSKYDFPGDD-------TPIIKGSAKLALEGDKGE 185

gi|52211250|emb|CAH37239.1| -AELLELVEMEVRELLSKYDFPGDD-------TPIIKGSAKLALEGDKGE 185

gi|53723856|ref|YP_104168.1| -AELLELVEMEVRELLSKYDFPGDD-------TPIIKGSAKLALEGDKGE 185

gi|16131810|ref|NP_418407.1| -EELLELVEMEVRELLSQYDFPGDD-------TPIVRGSALKALEGD--A 183

gi|31108|emb|CAA77750.1| FDAIMNFKKEETAKLIEKLDIKLDSEDKDKEGKPLLKAVMRRWLPAG-DA 349

:::: : *. :*:.: *: *. .*:::. * ..

gi|83721154|ref|YP_443574.1| LGEVAIMNLADALDT--------YIPTPER---------AVDGAFLMPVE 218

gi|52211250|emb|CAH37239.1| LGEVAIMNLADALDT--------YIPTPER---------AVDGAFLMPVE 218

gi|53723856|ref|YP_104168.1| LGEVAIMNLADALDT--------YIPTPER---------AVDGAFLMPVE 218

gi|16131810|ref|NP_418407.1| EWEAKILELAGFLDS--------YIPEPER---------AIDKPFLLPIE 216

gi|31108|emb|CAA77750.1| LLQMITIHLPSPVTAQKYRCELLYEGPPDDEAAMGIKSCDPKGPLMMYIS 399

: :.*.. : : * *: . .::: :.

gi|83721154|ref|YP_443574.1| DVFSISGRG-TVVTGRVERGVIKVGEEIEIVG------------------ 249

gi|52211250|emb|CAH37239.1| DVFSISGRG-TVVTGRVERGVIKVGEEIEIVG------------------ 249

gi|53723856|ref|YP_104168.1| DVFSISGRG-TVVTGRVERGVIKVGEEIEIVG------------------ 249

gi|16131810|ref|NP_418407.1| DVFSISGRG-TVVTGRVERGIIKVGEEVEIVG------------------ 247

gi|31108|emb|CAA77750.1| KMVPTSDKGRFYAFGRVFSGLVSTGLKVRIMGPNYTPGKKEDLYLKPIQR 449

.:.. *.:* . *** *::..* ::.*:*

gi|83721154|ref|YP_443574.1| --------IKATAKTTCT------GVEMFR-------------------- 265

gi|52211250|emb|CAH37239.1| --------IKATAKTTCT------GVEMFR-------------------- 265

gi|53723856|ref|YP_104168.1| --------IKATAKTTCT------GVEMFR-------------------- 265

gi|16131810|ref|NP_418407.1| --------IKETQKSTCT------GVEMFR-------------------- 263

gi|31108|emb|CAA77750.1| TILMMGRYVEPIEDVPCGNIVGLVGVDQFLVKTGTITTFEHAHNMRVMKF 499

:: . .* **: *

gi|83721154|ref|YP_443574.1| ------KLLDQGQAGDNVGILLRGTK---REDVERGQVLAKPG--SITPH 304

gi|52211250|emb|CAH37239.1| ------KLLDQGQAGDNVGILLRGTK---REDVERGQVLAKPG--SITPH 304

gi|53723856|ref|YP_104168.1| ------KLLDQGQAGDNVGILLRGTK---REDVERGQVLAKPG--SITPH 304

gi|16131810|ref|NP_418407.1| ------KLLDEGRAGENVGVLLRGIK---REEIERGQVLAKPG--TIKPH 302

gi|31108|emb|CAA77750.1| SVSPVVRVAVEAKNPADLPKLVEGLKRLAKSDPMVQCIIEESGEHIIAGA 549

:: :.: :: *:.* * :.: :: :.* *

gi|83721154|ref|YP_443574.1| THFTAEVYVLSKDEGGRHTPFFNN-----YRP------------------ 331

gi|52211250|emb|CAH37239.1| THFTAEVYVLSKDEGGRHTPFFNN-----YRP------------------ 331

gi|53723856|ref|YP_104168.1| THFTAEVYVLSKDEGGRHTPFFNN-----YRP------------------ 331

gi|16131810|ref|NP_418407.1| TKFESEVYILSKDEGGRHTPFFKG-----YRP------------------ 329

gi|31108|emb|CAA77750.1| GELHLEICLKDLEEDHACIPIKKSDPVVSYRETVSEESNVLCLSKSPNKH 599

.: *: : . :*. *: :. **

gi|83721154|ref|YP_443574.1| -QFYFRTTDVTGSIELPKDKEMVMPGDNVSITVKLIAP------------ 368

gi|52211250|emb|CAH37239.1| -QFYFRTTDVTGSIELPKDKEMVMPGDNVSITVKLIAP------------ 368

gi|53723856|ref|YP_104168.1| -QFYFRTTDVTGSIELPKDKEMVMPGDNVSITVKLIAP------------ 368

gi|16131810|ref|NP_418407.1| -QFYFRTTDVTGTIELPEGVEMVMPGDNIKMVVTLIHP------------ 366

gi|31108|emb|CAA77750.1| NRLYMKARPFPDGLAEDIDKGEVSARQELKQRARYLAEKYEWDVAEARKI 649

::*::: ... : . * . :::. . :

gi|83721154|ref|YP_443574.1| ---------------------------IAMEEGLRFAIRE---------- 381

gi|52211250|emb|CAH37239.1| ---------------------------IAMEEGLRFAIRE---------- 381

gi|53723856|ref|YP_104168.1| ---------------------------IAMEEGLRFAIRE---------- 381

gi|16131810|ref|NP_418407.1| ---------------------------IAMDDGLRFAIRE---------- 379

gi|31108|emb|CAA77750.1| WCFGPDGTGPNILTDITKGVQYLNEIKDSVVAGFQWATKEGALCEENMRG 699

:: *:::* :*

gi|83721154|ref|YP_443574.1| ------------------GGRTVGAG---VVAKIIE-------------- 396

gi|52211250|emb|CAH37239.1| ------------------GGRTVGAG---VVAKIIE-------------- 396

gi|53723856|ref|YP_104168.1| ------------------GGRTVGAG---VVAKIIE-------------- 396

gi|16131810|ref|NP_418407.1| ------------------GGRTVGAG---VVAKVLS-------------- 394

gi|31108|emb|CAA77750.1| VRFDVHDVTLHADAIHRGGGQIIPTARRCLYASVLTAQPRLMEPIYLVEI 749

**: : :. : *.::

gi|83721154|ref|YP_443574.1| --------------------------------------------------

gi|52211250|emb|CAH37239.1| --------------------------------------------------

gi|53723856|ref|YP_104168.1| --------------------------------------------------

gi|16131810|ref|NP_418407.1| --------------------------------------------------

gi|31108|emb|CAA77750.1| QCPEQVVGGIYGVLNRKRGHVFEESQVAGTPMFVVKAYLPVNESFGFTAD 799

gi|83721154|ref|YP_443574.1| --------------------------------------------------

gi|52211250|emb|CAH37239.1| --------------------------------------------------

gi|53723856|ref|YP_104168.1| --------------------------------------------------

gi|16131810|ref|NP_418407.1| --------------------------------------------------

gi|31108|emb|CAA77750.1| LRSNTGGQAFPQCVFDHWQILPGDPFDNSSRPSQVVAETRKRKGLKEGIP 849

gi|83721154|ref|YP_443574.1| ---------

gi|52211250|emb|CAH37239.1| ---------

gi|53723856|ref|YP_104168.1| ---------

gi|16131810|ref|NP_418407.1| ---------

gi|31108|emb|CAA77750.1| ALDNFLDKL 858

CLUSTAL 2.0.12 Multiple Sequence Alignments

Sequence format is Pearson

Sequence 1: gi|83721154|ref|YP_443574.1| 396 aa

Sequence 2: gi|52211250|emb|CAH37239.1| 396 aa

Sequence 3: gi|53723856|ref|YP_104168.1| 396 aa

Sequence 4: gi|16131810|ref|NP_418407.1| 394 aa

Sequence 5: gi|31108|emb|CAA77750.1| 858 aa

comparing

paramArg[setSeqNoRange]= off

comparing

Start of Pairwise alignments

Aligning...

Sequences (1:2) Aligned. Score: 100

Sequences (1:3) Aligned. Score: 100

Sequences (1:4) Aligned. Score: 80

Sequences (1:5) Aligned. Score: 17

Sequences (2:3) Aligned. Score: 100

Sequences (2:4) Aligned. Score: 80

Sequences (2:5) Aligned. Score: 17

Sequences (3:4) Aligned. Score: 80

Sequences (3:5) Aligned. Score: 17

Sequences (4:5) Aligned. Score: 20

Guide tree file created: [/ebi/extserv/clustalw-work/interactive/2010091006/clustalw2-20100910-0601057028.dnd]

There are 4 groups

Start of Multiple Alignment

Aligning...

Group 1: Sequences: 2 Score:8552

Group 2: Sequences: 3 Score:8552

Group 3: Sequences: 4 Score:7819

Group 4: Delayed

Alignment Score 14212

Alignment Score 45990

ITERATION 1 OF 1

Guide tree file created: [/tmp/filetR9Stj.dnd]

Start of Initial Alignment

Sequences:5 Score:3268

Start of Multiple Alignment

Aligning...

Sequences:5 Score:8232

Alignment Score 66513

Guide tree file created: [/tmp/file0N9eiB.dnd]

Start of Initial Alignment

Sequences:5 Score:3268

Start of Multiple Alignment

Aligning...

Sequences:5 Score:8232

Alignment Score 66513

Guide tree file created: [/tmp/fileRoJdDT.dnd]

Start of Initial Alignment

Sequences:5 Score:3268

Start of Multiple Alignment

Aligning...

Sequences:5 Score:8232

Alignment Score 66513

Guide tree file created: [/tmp/filecxVCuc.dnd]

Start of Initial Alignment

Sequences:5 Score:3133

Start of Multiple Alignment

Aligning...

Sequences:5 Score:7694

Alignment Score 62042

Guide tree file created: [/tmp/fileGjGfSv.dnd]

Start of Initial Alignment

Sequences:5 Score:2264

Start of Multiple Alignment

Aligning...

Sequences:5 Score:3591

Alignment Score 30286

Iteration improved Align score: 30286

FINAL score: 30286

Finished iteration

CLUSTAL-Alignment file created [/ebi/extserv/clustalw-work/interactive/2010091006/clustalw2-20100910-0601057028.aln]
