# Supplementary material for: Immunospecific Responses to Bacterial Elongation Factor Tu during Burkholderia Infection and Immunization
Source: PLoS One. 2010 Dec 17;5(12):e14361. doi: 10.1371/journal.pone.0014361 (PMC3003680; doi:10.1371/journal.pone.0014361)
Supplement: File S2 — Elongation factor Tu amino acid alignment and percent identity among five sequenced isolates of B. pseudomallei. (0.03 MB DOCX) [file pone.0014361.s002.docx]

**elongation factor Tu [Burkholderia pseudomallei K96243]**

GenBank: CAH37239.1

>gi|52211250|emb|CAH37239.1| elongation factor Tu [Burkholderia pseudomallei K96243]

MAKEKFERTKPHVNVGTIGHVDHGKTTLTAAIATVLSAKFGGEAKKYDEIDAAPEEKARGITINTAHIEY

ETANRHYAHVDCPGHADYVKNMITGAAQMDGAILVCSAADGPMPQTREHILLARQVGVPYIIVFLNKCDM

VDDAELLELVEMEVRELLSKYDFPGDDTPIIKGSAKLALEGDKGELGEVAIMNLADALDTYIPTPERAVD

GAFLMPVEDVFSISGRGTVVTGRVERGVIKVGEEIEIVGIKATAKTTCTGVEMFRKLLDQGQAGDNVGIL

LRGTKREDVERGQVLAKPGSITPHTHFTAEVYVLSKDEGGRHTPFFNNYRPQFYFRTTDVTGSIELPKDK

EMVMPGDNVSITVKLIAPIAMEEGLRFAIREGGRTVGAGVVAKIIE

Uniprot/Swiss prot # Q63PZ6

# translation elongation factor Tu [Burkholderia pseudomallei Pasteur 52237]

NCBI Reference Sequence: ZP_04896797.1

[GenPept](http://www.ncbi.nlm.nih.gov/protein/254190289?report=genpept) [Graphics](http://www.ncbi.nlm.nih.gov/protein/254190289?report=graph)

>gi|254190289|ref|ZP_04896797.1| translation elongation factor Tu [Burkholderia pseudomallei Pasteur 52237]

MAKEKFERTKPHVNVGTIGHVDHGKTTLTAAIATVLSAKFGGEAKKYDEIDAAPEEKARGITINTAHIEY

ETANRHYAHVDCPGHADYVKNMITGAAQMDGAILVCSAADGPMPQTREHILLARQVGVPYIIVFLNKCDM

VDDAELLELVEMEVRELLSKYDFPGDDTPIIKGSAKLALEGDKGELGEVAIMNLADALDTYIPTPERAVD

GAFLMPVEDVFSISGRGTVVTGRVERGVIKVGEEIEIVGIKATAKTTCTGVEMFRKLLDQGQAGDNVGIL

LRGTKREDVERGQVLAKPGSITPHTHFTAEVYVLSKDEGGRHTPFFNNYRPQFYFRTTDVTGSIELPKDK

EMVMPGDNVSITVKLIAPIAMEEGLRFAIREGGRTVGAGVVAKIIE

# translation elongation factor Tu [Burkholderia pseudomallei 406e]

NCBI Reference Sequence: ZP_04968012.1

[GenPept](http://www.ncbi.nlm.nih.gov/protein/254300567?report=genpept) [Graphics](http://www.ncbi.nlm.nih.gov/protein/254300567?report=graph)

>gi|254300567|ref|ZP_04968012.1| translation elongation factor Tu [Burkholderia pseudomallei 406e]

MAKEKFERTKPHVNVGTIGHVDHGKTTLTAAIATVLSAKFGGEAKKYDEIDAAPEEKARGITINTAHIEY

ETANRHYAHVDCPGHADYVKNMITGAAQMDGAILVCSAADGPMPQTREHILLARQVGVPYIIVFLNKCDM

VDDAELLELVEMEVRELLSKYDFPGDDTPIIKGSAKLALEGDKGELGEVAIMNLADALDTYIPTPERAVD

GAFLMPVEDVFSISGRGTVVTGRVERGVIKVGEEIEIVGIKATAKTTCTGVEMFRKLLDQGQAGDNVGIL

LRGTKREDVERGQVLAKPGSITPHTHFTAEVYVLSKDEGGRHTPFFNNYRPQFYFRTTDVTGSIELPKDK

EMVMPGDNVSITVKLIAPIAMEEGLRFAIREGGRTVGAGVVAKIIE

# translation elongation factor Tu [Burkholderia pseudomallei 1106a]

GenBank: ABN92409.1

[GenPept](http://www.ncbi.nlm.nih.gov/protein/126228869?report=genpept) [Graphics](http://www.ncbi.nlm.nih.gov/protein/126228869?report=graph)

>gi|126228869|gb|ABN92409.1| translation elongation factor Tu [Burkholderia pseudomallei 1106a]

MAKEKFERTKPHVNVGTIGHVDHGKTTLTAAIATVLSAKFGGEAKKYDEIDAAPEEKARGITINTAHIEY

ETANRHYAHVDCPGHADYVKNMITGAAQMDGAILVCSAADGPMPQTREHILLARQVGVPYIIVFLNKCDM

VDDAELLELVEMEVRELLSKYDFPGDDTPIIKGSAKLALEGDKGELGEVAIMNLADALDTYIPTPERAVD

GAFLMPVEDVFSISGRGTVVTGRVERGVIKVGEEIEIVGIKATAKTTCTGVEMFRKLLDQGQAGDNVGIL

LRGTKREDVERGQVLAKPGSITPHTHFTAEVYVLSKDEGGRHTPFFNNYRPQFYFRTTDVTGSIELPKDK

EMVMPGDNVSITVKLIAPIAMEEGLRFAIREGGRTVGAGVVAKIIE

# translation elongation factor Tu [Burkholderia pseudomallei MSHR346]

NCBI Reference Sequence: YP_002898614.1

[GenPept](http://www.ncbi.nlm.nih.gov/protein/237814163?report=genpept) [Graphics](http://www.ncbi.nlm.nih.gov/protein/237814163?report=graph)

>gi|237814163|ref|YP_002898614.1| translation elongation factor Tu [Burkholderia pseudomallei MSHR346]

MAKEKFERTKPHVNVGTIGHVDHGKTTLTAAIATVLSAKFGGEAKKYDEIDAAPEEKARGITINTAHIEY

ETANRHYAHVDCPGHADYVKNMITGAAQMDGAILVCSAADGPMPQTREHILLARQVGVPYIIVFLNKCDM

VDDAELLELVEMEVRELLSKYDFPGDDTPIIKGSAKLALEGDKGELGEVAIMNLADALDTYIPTPERAVD

GAFLMPVEDVFSISGRGTVVTGRVERGVIKVGEEIEIVGIKATAKTTCTGVEMFRKLLDQGQAGDNVGIL

LRGTKREDVERGQVLAKPGSITPHTHFTAEVYVLSKDEGGRHTPFFNNYRPQFYFRTTDVTGSIELPKDK

EMVMPGDNVSITVKLIAPIAMEEGLRFAIREGGRTVGAGVVAKIIE

CLUSTAL 2.0.12 multiple sequence alignment

gi|52211250|emb|CAH37239.1| MAKEKFERTKPHVNVGTIGHVDHGKTTLTAAIATVLSAKFGGEAKKYDEI 50

gi|254190289|ref|ZP_04896797.1 MAKEKFERTKPHVNVGTIGHVDHGKTTLTAAIATVLSAKFGGEAKKYDEI 50

gi|254300567|ref|ZP_04968012.1 MAKEKFERTKPHVNVGTIGHVDHGKTTLTAAIATVLSAKFGGEAKKYDEI 50

gi|126228869|gb|ABN92409.1| MAKEKFERTKPHVNVGTIGHVDHGKTTLTAAIATVLSAKFGGEAKKYDEI 50

gi|237814163|ref|YP_002898614. MAKEKFERTKPHVNVGTIGHVDHGKTTLTAAIATVLSAKFGGEAKKYDEI 50

**************************************************

gi|52211250|emb|CAH37239.1| DAAPEEKARGITINTAHIEYETANRHYAHVDCPGHADYVKNMITGAAQMD 100

gi|254190289|ref|ZP_04896797.1 DAAPEEKARGITINTAHIEYETANRHYAHVDCPGHADYVKNMITGAAQMD 100

gi|254300567|ref|ZP_04968012.1 DAAPEEKARGITINTAHIEYETANRHYAHVDCPGHADYVKNMITGAAQMD 100

gi|126228869|gb|ABN92409.1| DAAPEEKARGITINTAHIEYETANRHYAHVDCPGHADYVKNMITGAAQMD 100

gi|237814163|ref|YP_002898614. DAAPEEKARGITINTAHIEYETANRHYAHVDCPGHADYVKNMITGAAQMD 100

**************************************************

gi|52211250|emb|CAH37239.1| GAILVCSAADGPMPQTREHILLARQVGVPYIIVFLNKCDMVDDAELLELV 150

gi|254190289|ref|ZP_04896797.1 GAILVCSAADGPMPQTREHILLARQVGVPYIIVFLNKCDMVDDAELLELV 150

gi|254300567|ref|ZP_04968012.1 GAILVCSAADGPMPQTREHILLARQVGVPYIIVFLNKCDMVDDAELLELV 150

gi|126228869|gb|ABN92409.1| GAILVCSAADGPMPQTREHILLARQVGVPYIIVFLNKCDMVDDAELLELV 150

gi|237814163|ref|YP_002898614. GAILVCSAADGPMPQTREHILLARQVGVPYIIVFLNKCDMVDDAELLELV 150

**************************************************

gi|52211250|emb|CAH37239.1| EMEVRELLSKYDFPGDDTPIIKGSAKLALEGDKGELGEVAIMNLADALDT 200

gi|254190289|ref|ZP_04896797.1 EMEVRELLSKYDFPGDDTPIIKGSAKLALEGDKGELGEVAIMNLADALDT 200

gi|254300567|ref|ZP_04968012.1 EMEVRELLSKYDFPGDDTPIIKGSAKLALEGDKGELGEVAIMNLADALDT 200

gi|126228869|gb|ABN92409.1| EMEVRELLSKYDFPGDDTPIIKGSAKLALEGDKGELGEVAIMNLADALDT 200

gi|237814163|ref|YP_002898614. EMEVRELLSKYDFPGDDTPIIKGSAKLALEGDKGELGEVAIMNLADALDT 200

**************************************************

gi|52211250|emb|CAH37239.1| YIPTPERAVDGAFLMPVEDVFSISGRGTVVTGRVERGVIKVGEEIEIVGI 250

gi|254190289|ref|ZP_04896797.1 YIPTPERAVDGAFLMPVEDVFSISGRGTVVTGRVERGVIKVGEEIEIVGI 250

gi|254300567|ref|ZP_04968012.1 YIPTPERAVDGAFLMPVEDVFSISGRGTVVTGRVERGVIKVGEEIEIVGI 250

gi|126228869|gb|ABN92409.1| YIPTPERAVDGAFLMPVEDVFSISGRGTVVTGRVERGVIKVGEEIEIVGI 250

gi|237814163|ref|YP_002898614. YIPTPERAVDGAFLMPVEDVFSISGRGTVVTGRVERGVIKVGEEIEIVGI 250

**************************************************

gi|52211250|emb|CAH37239.1| KATAKTTCTGVEMFRKLLDQGQAGDNVGILLRGTKREDVERGQVLAKPGS 300

gi|254190289|ref|ZP_04896797.1 KATAKTTCTGVEMFRKLLDQGQAGDNVGILLRGTKREDVERGQVLAKPGS 300

gi|254300567|ref|ZP_04968012.1 KATAKTTCTGVEMFRKLLDQGQAGDNVGILLRGTKREDVERGQVLAKPGS 300

gi|126228869|gb|ABN92409.1| KATAKTTCTGVEMFRKLLDQGQAGDNVGILLRGTKREDVERGQVLAKPGS 300

gi|237814163|ref|YP_002898614. KATAKTTCTGVEMFRKLLDQGQAGDNVGILLRGTKREDVERGQVLAKPGS 300

**************************************************

gi|52211250|emb|CAH37239.1| ITPHTHFTAEVYVLSKDEGGRHTPFFNNYRPQFYFRTTDVTGSIELPKDK 350

gi|254190289|ref|ZP_04896797.1 ITPHTHFTAEVYVLSKDEGGRHTPFFNNYRPQFYFRTTDVTGSIELPKDK 350

gi|254300567|ref|ZP_04968012.1 ITPHTHFTAEVYVLSKDEGGRHTPFFNNYRPQFYFRTTDVTGSIELPKDK 350

gi|126228869|gb|ABN92409.1| ITPHTHFTAEVYVLSKDEGGRHTPFFNNYRPQFYFRTTDVTGSIELPKDK 350

gi|237814163|ref|YP_002898614. ITPHTHFTAEVYVLSKDEGGRHTPFFNNYRPQFYFRTTDVTGSIELPKDK 350

**************************************************

gi|52211250|emb|CAH37239.1| EMVMPGDNVSITVKLIAPIAMEEGLRFAIREGGRTVGAGVVAKIIE 396

gi|254190289|ref|ZP_04896797.1 EMVMPGDNVSITVKLIAPIAMEEGLRFAIREGGRTVGAGVVAKIIE 396

gi|254300567|ref|ZP_04968012.1 EMVMPGDNVSITVKLIAPIAMEEGLRFAIREGGRTVGAGVVAKIIE 396

gi|126228869|gb|ABN92409.1| EMVMPGDNVSITVKLIAPIAMEEGLRFAIREGGRTVGAGVVAKIIE 396

gi|237814163|ref|YP_002898614. EMVMPGDNVSITVKLIAPIAMEEGLRFAIREGGRTVGAGVVAKIIE 396

**********************************************

Score = 40

Length of alignment = 1

Sequence gi|254190289|ref|ZP_04896797.1 : 1 - 396 (Sequence length = 1)

Sequence gi|52211250|emb|CAH37239.1| : 1 - 396 (Sequence length = 1)

gi|254190289|ref|ZP_04896797.1 V

|

gi|52211250|emb|CAH37239.1| V

Percentage ID = 100.00

Score = 40

Length of alignment = 1

Sequence gi|254300567|ref|ZP_04968012.1 : 1 - 396 (Sequence length = 1)

Sequence gi|52211250|emb|CAH37239.1| : 1 - 396 (Sequence length = 1)

gi|254300567|ref|ZP_04968012.1 V

|

gi|52211250|emb|CAH37239.1| V

Percentage ID = 100.00

Score = 40

Length of alignment = 1

Sequence gi|254300567|ref|ZP_04968012.1 : 1 - 396 (Sequence length = 1)

Sequence gi|254190289|ref|ZP_04896797.1 : 1 - 396 (Sequence length = 1)

gi|254300567|ref|ZP_04968012.1 V

|

gi|254190289|ref|ZP_04896797.1 V

Percentage ID = 100.00

Score = 40

Length of alignment = 1

Sequence gi|126228869|gb|ABN92409.1| : 1 - 396 (Sequence length = 1)

Sequence gi|52211250|emb|CAH37239.1| : 1 - 396 (Sequence length = 1)

gi|126228869|gb|ABN92409.1| V

|

gi|52211250|emb|CAH37239.1| V

Percentage ID = 100.00

Score = 40

Length of alignment = 1

Sequence gi|126228869|gb|ABN92409.1| : 1 - 396 (Sequence length = 1)

Sequence gi|254190289|ref|ZP_04896797.1 : 1 - 396 (Sequence length = 1)

gi|126228869|gb|ABN92409.1| V

|

gi|254190289|ref|ZP_04896797.1 V

Percentage ID = 100.00

Score = 40

Length of alignment = 1

Sequence gi|126228869|gb|ABN92409.1| : 1 - 396 (Sequence length = 1)

Sequence gi|254300567|ref|ZP_04968012.1 : 1 - 396 (Sequence length = 1)

gi|126228869|gb|ABN92409.1| V

|

gi|254300567|ref|ZP_04968012.1 V

Percentage ID = 100.00

Score = 40

Length of alignment = 1

Sequence gi|237814163|ref|YP_002898614. : 1 - 396 (Sequence length = 1)

Sequence gi|52211250|emb|CAH37239.1| : 1 - 396 (Sequence length = 1)

gi|237814163|ref|YP_002898614. V

|

gi|52211250|emb|CAH37239.1| V

Percentage ID = 100.00

Score = 40

Length of alignment = 1

Sequence gi|237814163|ref|YP_002898614. : 1 - 396 (Sequence length = 1)

Sequence gi|254190289|ref|ZP_04896797.1 : 1 - 396 (Sequence length = 1)

gi|237814163|ref|YP_002898614. V

|

gi|254190289|ref|ZP_04896797.1 V

Percentage ID = 100.00

Score = 40

Length of alignment = 1

Sequence gi|237814163|ref|YP_002898614. : 1 - 396 (Sequence length = 1)

Sequence gi|254300567|ref|ZP_04968012.1 : 1 - 396 (Sequence length = 1)

gi|237814163|ref|YP_002898614. V

|

gi|254300567|ref|ZP_04968012.1 V

Percentage ID = 100.00

Score = 40

Length of alignment = 1

Sequence gi|237814163|ref|YP_002898614. : 1 - 396 (Sequence length = 1)

Sequence gi|126228869|gb|ABN92409.1| : 1 - 396 (Sequence length = 1)

gi|237814163|ref|YP_002898614. V

|

gi|126228869|gb|ABN92409.1| V

Percentage ID = 100.00
