# Supplementary figures and images for: Immunospecific Responses to Bacterial Elongation Factor Tu during Burkholderia Infection and Immunization
Source: PLoS One. 2010 Dec 17;5(12):e14361. doi: 10.1371/journal.pone.0014361 (PMC3003680; doi:10.1371/journal.pone.0014361)

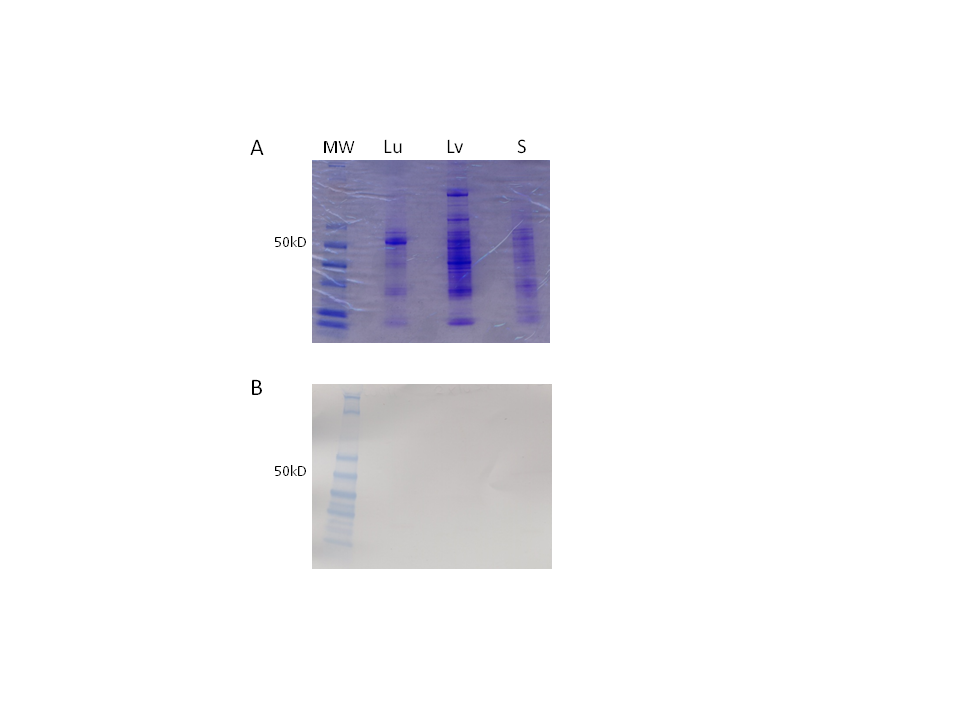

Supplement: File S3 — Antibody against bacterial EF-Tu does not react with mammalian tissue. (A) Coomassie stained gel of mouse lung (Lu), liver (Lv), and spleen (S) homogenates. MW = SeeBlue plus2 molecular weight ladder. (B) Western blot of mouse tissues using 1∶100 dilution of affinity purified EF-Tu IgG. (0.24 MB TIF) [file pone.0014361.s003.tif]
